# Supplementary material for: Neoadjuvant anti-OX40 (MEDI6469) therapy in patients with head and neck squamous cell carcinoma activates and expands antigen-specific tumor-infiltrating T cells
Source: Nat Commun. 2021 Feb 16;12:1047. doi: 10.1038/s41467-021-21383-1 (PMC7886909; doi:10.1038/s41467-021-21383-1)
Supplement: Supplementary file 1 — Supplementary Information [file 41467_2021_21383_MOESM1_ESM.pdf]

Neoadjuvant anti-OX40 (MEDI6469) therapy in patients with head and neck squamous cell carcinoma activates and expands antigen-specific tumor-infiltrating T cells

Duhen et al., 2020

SUPPLEMENTARY FIGURES

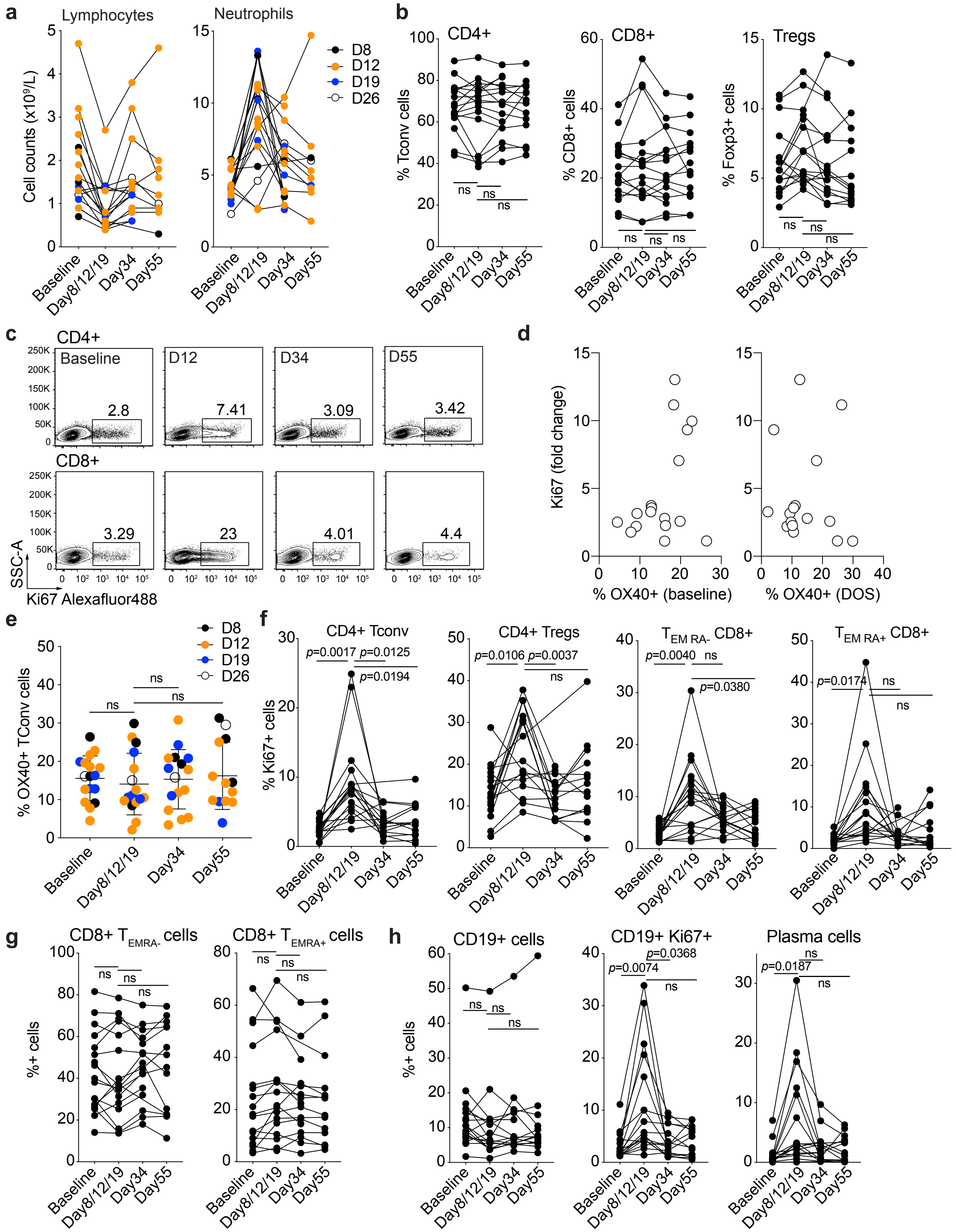

Supplementary Figure 1. Immune activation in patients after OX40 administration
a, Blood lymphocyte and neutrophil counts were assessed at baseline, day of surgery (D8, D12, D19 and D26), D34 and D55. Individual patients are shown. **b**, Percentages of CD4+ Tconv, CD8+ and CD4+ Treg cells in each patient during and after anti-OX40 administration as assessed by flow cytometry. **c**, Representative expression of Ki-67 in CD4+ and CD8+ T cells at different timepoints before and after OX40 administration. **d**, Comparison of the expression of OX40 at baseline and DOS with the fold increase in Ki67 in CD4+ Tconv cells. **e,** Expression of OX40 on CD4+ Tconv cells at all timepoints before and after treatment. Error bars indicate mean ± SEM. **f**, Percentages of Ki-67 expression in each patient during OX40 treatment in CD4+ Tconv memory cells, CD4+ Treg cells, CD8+ T_{EMRA-} and T_{EMRA+} cells. **g**, Percentages of CD8+ T_{EMRA-} and CD8+ T_{EMRA+} cells in each patient during and after anti-OX40 administration. **h**, Percentages of total CD19+ cells, Ki-67+ CD19+ cells and plasmablasts, identified by absence of IgD and expression of CD38, in each patient. *$p < 0.05$; **$p < 0.01$; ***$p < 0.001$; ****$p < 0.0001$; ns, not significant. *p*-values were determined using the mixed-effects model and Tukey multiple comparison test in all patients (**b, e-h**). $N = 3$ patients in the D8 cohort, $N = 9$ patients in the D12 cohort, $N = 4$ patients in the D19 cohort and $N = 1$ patients in the D26 cohort (**a,b and d-h**). In **a** and **e**, black circles represent the D8, orange circles the D12 and blue circles the D19 cohort, the D26 patient is represented by an open circle.

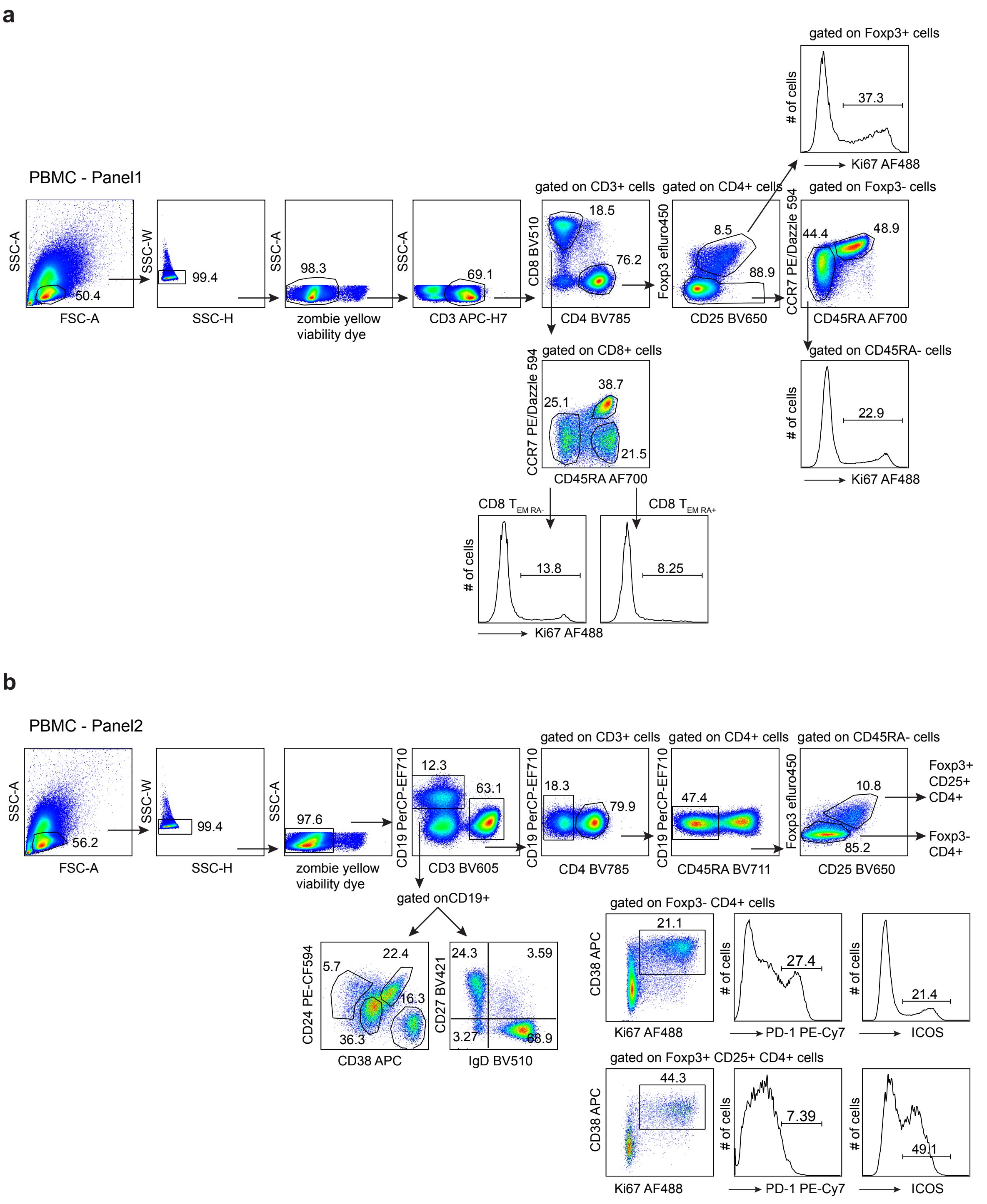

Supplementary Figure 2. Gating strategies for PBMC phenotyping

a,**b**, Gating strategy to analyze the phenotype of PBMC subsets before and after anti-OX40 administration (Fig. 1 and Supplementary Fig. 1). **a**, Panel1, **b**, Panel2.

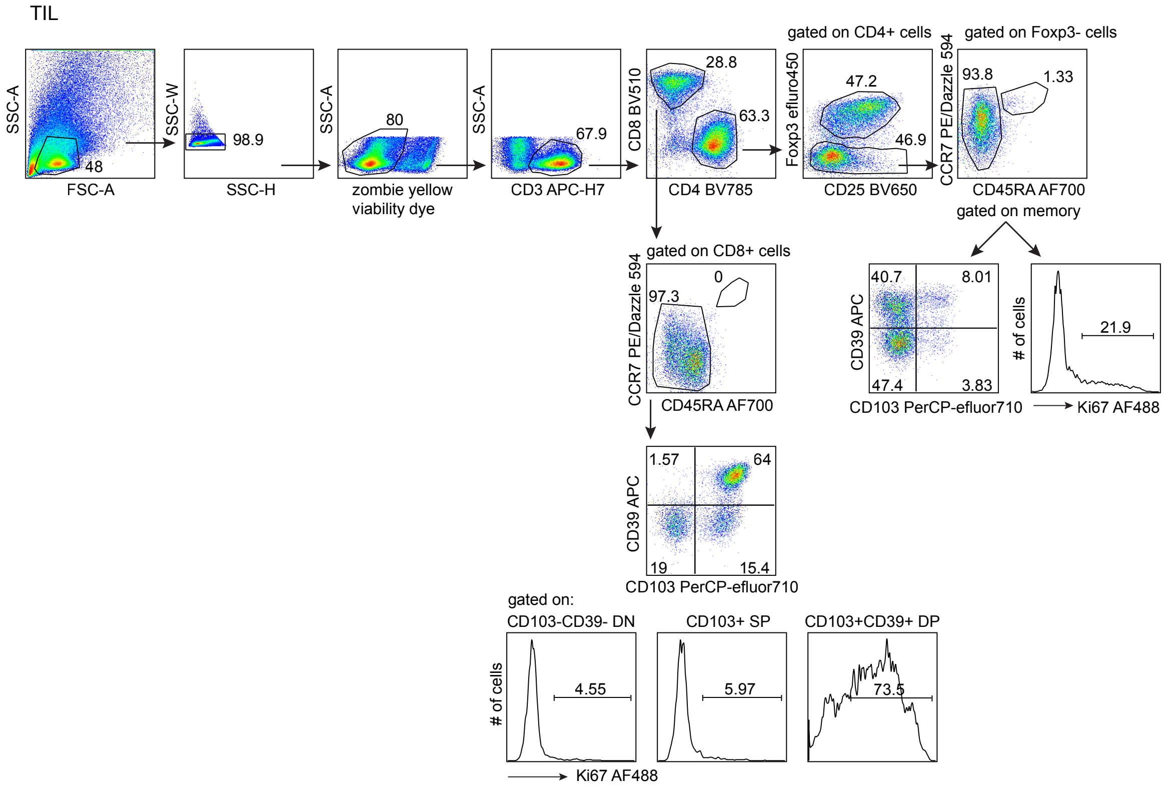

Supplementary Figure 3. **Gating strategy for TIL**

Gating strategy to analyze the phenotype of CD8+ TIL before and after administration of anti-OX40 (Fig. 2).

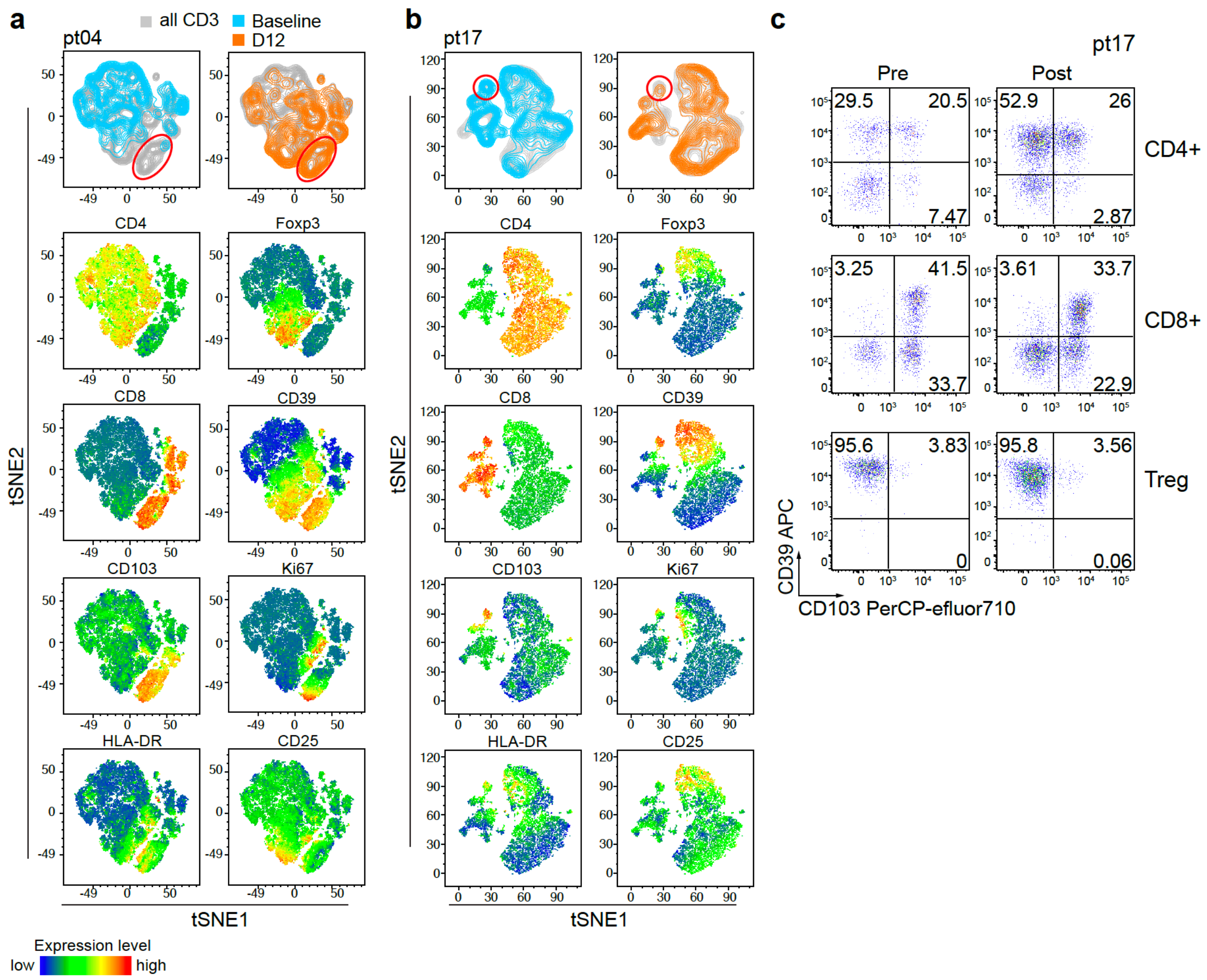

Supplementary Figure 4. tSNE analysis in TIL pre and post OX40

a,**b**, tSNE analysis of the biopsy and DOS specimen from patients HNOX04 (**a**) and HNOX17 (**b**). Only CD3+ cells were included in the analysis. Blue represents the baseline sample, orange the day of surgery sample and grey is the concatenated file. The red circle identifies a population of cells expressing both CD103 and CD39. **c**, Flow cytometric analysis of the expression of CD103 and CD39 in CD4+, CD8+ and CD4+ Treg cells in a non-responding HNSCC patient pre and post OX40 therapy.

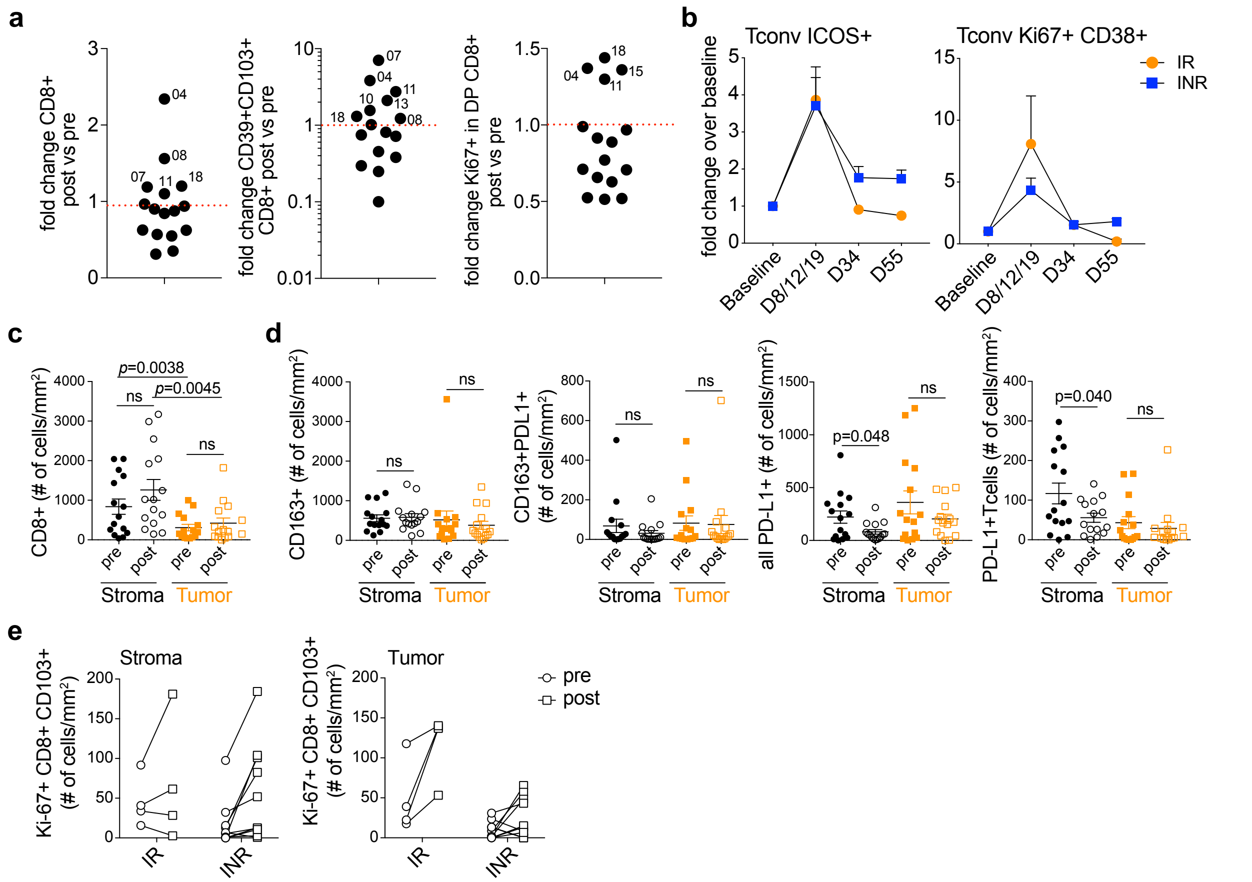

Supplementary Figure 5. Comparison of multiplex IHC with flow cytometric analysis
a, Analysis of the fold change of CD8+ T cells, CD103+ CD39+ cells and Ki-67+ cells among TIL in all HNOX patients. **b**, Summary of the fold change in ICOS and Ki67/CD38 expression during OX40 treatment in CD4+ Tconv memory cells grouped as immune responders (IR) (*N* = 4, orange) or immune non-responder (INR) (*N* = 13, blue) status. The mean + SEM is shown for IR and INR. **c**, Summary of the number of total CD8+ T cells among tumor and stroma in patients pre and post OX40. **d**, Summary of the number of total CD163+ cells, CD163+ PD-L1+ cells, total PD-L1+ cells and PD-L1+ CD3+ T cells among tumor and stroma in patients pre and post OX40. Error bars indicate mean ± SEM. *$p < 0.05$; **$p < 0.01$; ***$p < 0.001$; ****$p < 0.0001$; ns, not significant. *p*-values were determined by paired two-tailed Student's t-test between pre and post samples (**c, d**) and between tumor and stroma (**c**). **e**, Correlation of pre and post samples for the expression of Ki-67, CD103 and CD8 in tumor and stroma. *$p < 0.05$ were considered significant; p-values were determined by paired two-tailed Student's t-test between pre and post samples.

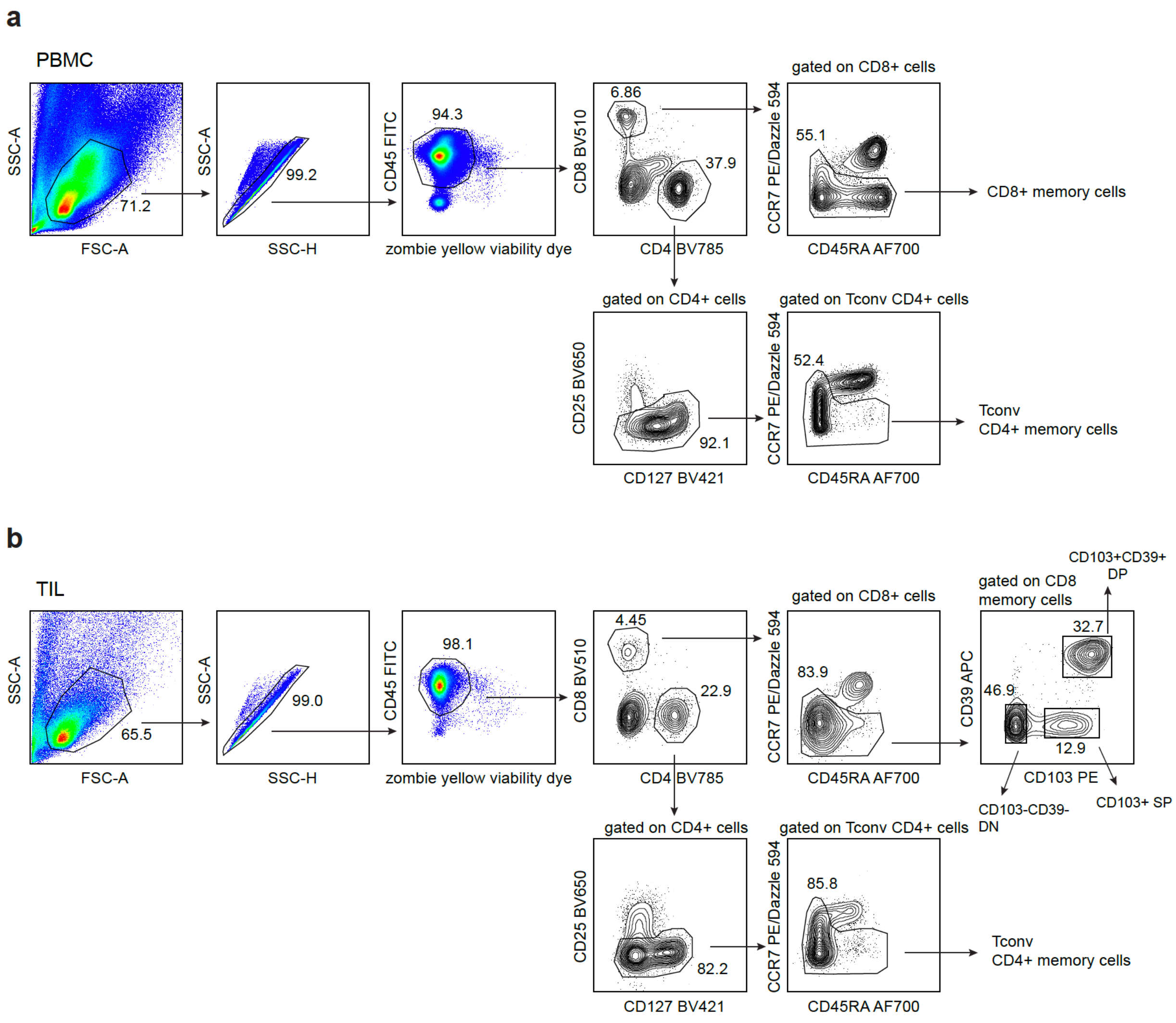

Supplementary Figure 6. Gating strategy for T cell expansion and TCRβ sequencing
a, Gating strategy to sort memory CD4+ and CD8+ T cells from peripheral blood for TCR repertoire analysis (Fig. 5). **b**, Gating strategy to sort DN, SP and DP CD8+ T cells and memory CD4+ T cells from the primary tumor pre and post anti-OX40 administration for TCR repertoire analysis and T cell expansion for neoantigen reactivity screening (Fig. 5 and Fig. 6).

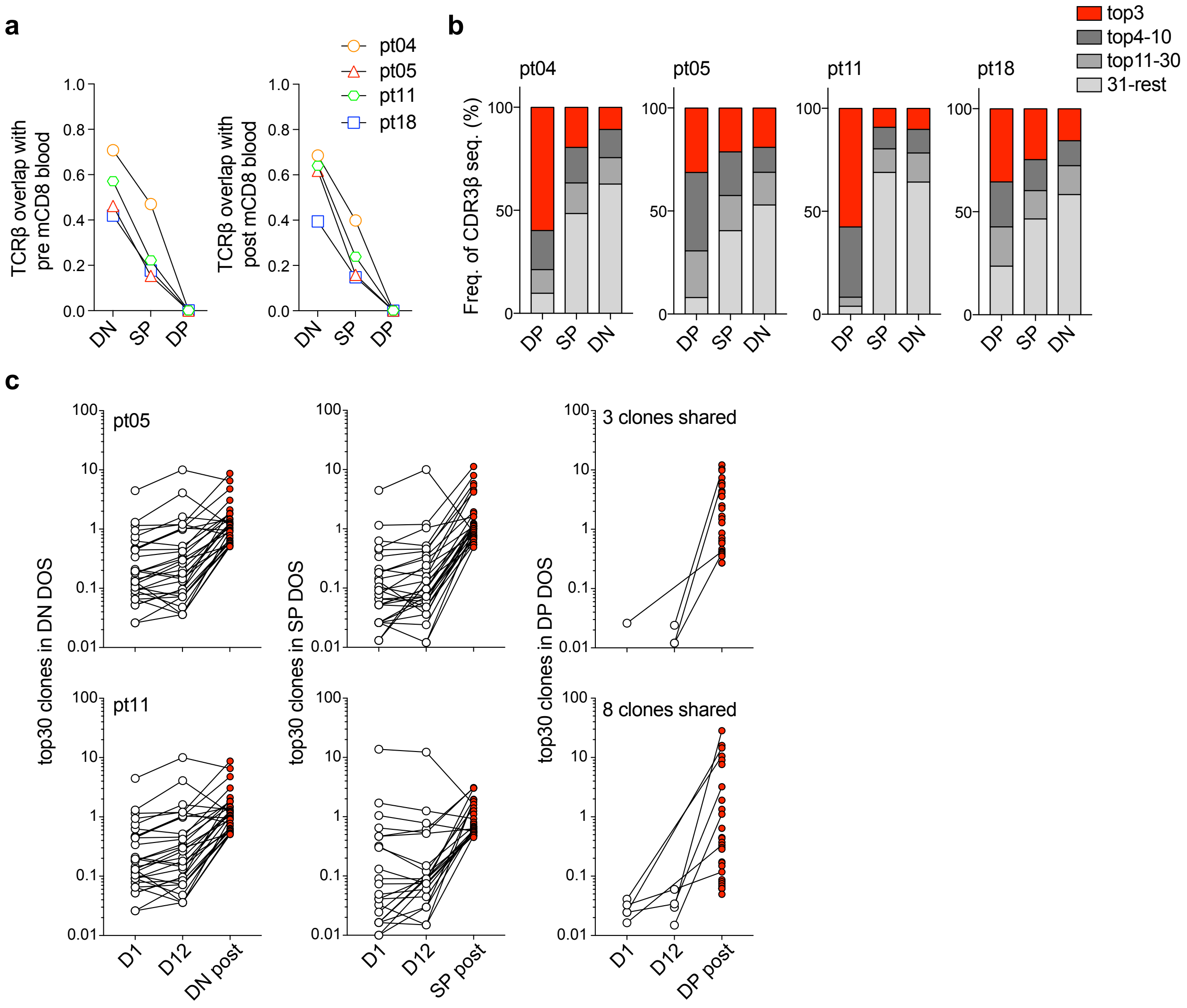

Supplementary Figure 7. TCRβ sequencing analysis

a, Similarity between the TCRβ repertoires (overlap) was calculated using the Morisita-Horn index. The TCRβ repertoire of memory CD8+ T cells in peripheral blood before and after anti-OX40 was compared with the TCR repertoire of DN, SP and DP CD8+ TIL subsets. Colors and symbols depict the 4 patients. **b**, Diversity of the TCRβ repertoire within DN, SP, and DP CD8+ TIL. The frequencies of the 1st to 3rd most frequent (red), 4th to 10th most frequent, the 11th to 30th most frequent, and the rest of the clonotypes (others) are shown for HNOX04, HNOX05, HNOX11 and HNOX18. **c**, The top 30 clones in the DOS specimen (DN, SP and DP) were separately compared to the same subset pre-treatment and to memory CD8+ T cells in blood at D1 and D12. Black open circles represent the blood before and after anti-OX40, red filled circles represent the DOS (post) TIL specimen. Connecting lines indicate the presence of the same TCRβ sequence in each subset. In **b-f** *N* = 4 patients (HNOX04, HNOX05, HNOX11 and HNOX18) were analyzed.

Supplementary Table 1a. Individual patients, cohorts and clinical characteristics

Patient ID	Age	Stage AJCC 7th	Stage AJCC 8th	Tumor site	HPV status	Treatment cohort
HNOX01	67	III	III	Oropharynx	neg	D-26
HNOX03	61	IV-A	I	Oropharynx	pos	D-8
HNOX04	54	IV-A	IV-A	Hypopharynx	neg	D-12
HNOX05	53	III	III	Oral	neg	D-19
HNOX06	63	III	III	Oropharynx	neg	D-8
HNOX07	54	II	III	Oral	neg	D-19
HNOX08	61	III	III	Oral	neg	D-12
HNOX09	63	IV-A	II	Oropharynx	pos	D-19
HNOX10	67	IV-A	IV-A	Oral	neg	D-8
HNOX11	68	IV-A	IV-A	Oral	neg	D-12
HNOX13	50	III	I	Oropharynx	pos	D-12
HNOX14	61	III	III	Oropharynx	neg	D-12
HNOX15	49	IV-A	I	Oropharynx	pos	D-12
HNOX16	69	IV	II	Oropharynx	pos	D-19
HNOX17	44	IV-A	IV-A	Larynx glottic	neg	D-12
HNOX18	57	IV-A	II	Oropharynx	pos	D-12
HNOX19	80	Iva	II	Oral	neg	D-12

AJCC, American Joint Committee on Cancer
HPV, Human Papilloma virus

Supplementary Table 1b. Patient and clinical characteristics – summary

**Characteristic**	
Age (years), Mean ± SD	60 ± 9
Sex, n (%)	
Men	13 (76)
Women	4 (24)
HPV status, n	
positive	6
negative	11
AJCC (7th Edition) clinical stage, n (%)	
II	2 (12)
III	5 (29)
IV/IV-A	10 (59)
AJCC (8th Edition) clinical stage, n (%)	
I	3 (18)
II	4 (23.5)
III	6 (35)
IV/IV-A	4 (23.5)
Site, n (%)	
Oral	6 (35)
Oropharynx	9 (53)
Hypopharynx	1 (6)
Larynx	1 (6)
Prior Therapy, n (%)	
Treatment naïve	14 (82)
Radiation Therapy	3 (18)

AJCC, American Joint Committee on Cancer
HPV, Human Papilloma virus

Supplementary Table 2. Adverse events related to OX40, *N*=17

**Averse Event**	**Any Grade (n)**	**%**	**Grade 3 (n)**	**%**	**Grade 4 (n)**	**%**
Anorexia	1	(5.88)	0	(0)	0	(0)
Anxiety	2	(11.76)	0	(0)	0	(0)
Arthralgia	1	(5.88)	0	(0)	0	(0)
Atrial fibrillation	1	(5.88)	0	(0)	0	(0)
Bacteremia	1	(5.88)	1	(5.88)	0	(0)
Bradycardia	1	(5.88)	0	(0)	0	(0)
Cardiac troponin increased	1	(5.88)	0	(0)	0	(0)
Clostridium difficile	1	(5.88)	0	(0)	0	(0)
Cellulitis	1	(5.88)	0	(0)	0	(0)
Chills	2	(11.76)	0	(0)	0	(0)
Dehydration	2	(5.88)	0	(0)	0	(0)
Diarrhea	1	(5.88)	0	(0)	0	(0)
Fatigue	3	(11.76)	0	(0)	0	(0)
Fever	3	(11.76)	0	(0)	0	(0)
Granulation tissue at forearm donor site	1	(5.88)	0	(0)	0	(0)
Headache	2	(5.88)	0	(0)	0	(0)
Hyperglycemia	3	(17.65)	0	(0)	0	(0)
Hypocalcemia	2	(11.76)	0	(0)	0	(0)
Hypokalemia	1	(5.88)	1	(5.88)	0	(0)
Hypomagnesemia	2	(11.76)	0	(0)	0	(0)
Hyponatremia	5	(29.41)	1	(5.88)	0	(0)
Hypophosphatemia	1	(5.88)	0	(0)	0	(0)
Hypovolemia	1	(5.88)	0	(0)	0	(0)
Increasing swelling	1	(5.88)	0	(0)	0	(0)
Insomnia	1	(5.88)	0	(0)	0	(0)
Left eye pain	1	(5.88)	0	(0)	0	(0)
Leg cramps	1	(5.88)	0	(0)	0	(0)
Localized edema (tumor injection site)	1	(5.88)	0	(0)	0	(0)
Low platelet count	1	(5.88)	0	(0)	0	(0)
Lymphocytes, Dec	1	(5.88)	0	(0)	0	(0)
Malaise	3	(5.88)	0	(0)	0	(0)
Mouth tingling sensation	1	(5.88)	0	(0)	0	(0)
Myalgia	1	(5.88)	0	(0)	0	(0)
Nausea	7	(41.17)	0	(0)	0	(0)
Neck stiffness	1	(5.88)	0	(0)	0	(0)
Oral pain (soreness of tongue)	1	(5.88)	0	(0)	0	(0)
Rash	1	(5.88)	0	(0)	0	(0)

Sepsis	1	(5.88)	0	(0)	1	(5.88)
Shortness of breath	1	(5.88)	0	(0)	0	(0)
Tired	2	(5.88)	0	(0)	0	(0)
Weakness	1	(5.88)	0	(0)	0	(0)
Weight loss	1	(5.88)	0	(0)	0	(0)
Worsening hot flashes	1	(5.88)	0	(0)	0	(0)

Supplementary Table 3. Adverse events related to surgery, *N*=17

Averse Event	Any Grade (n)	%	Grade III (n)	%	Grade IV (n)	%
Abdominal Fullness	2	(11.76)	0	(0)	0	(0)
Acute respiratory failure	2	(11.76)	0	(0)	0	(0)
Anxiety	4	(23.53)	0	(0)	0	(0)
Bacteremia	1	(5.88)	0	(0)	1	(5.88)
Bradycardia	1	(5.88)	0	(0)	0	(0)
Clostridium difficile	1	(5.88)	0	(0)	0	(0)
Cellulitis	1	(5.88)	1	(5.88)	0	(0)
Chest pain	1	(5.88)	0	(0)	0	(0)
Constipation	3	(17.65)	0	(0)	0	(0)
Coughing	2	(11.76)	0	(0)	0	(0)
Dehydration	2	(11.76)	0	(0)	0	(0)
Adventitious breath sounds	3	(11.76)	0	(0)	0	(0)
Difficulty breathing/SOB	3	(17.65)	0	(0)	0	(0)
Dizziness	1	(5.88)	0	(0)	0	(0)
Dysphagia	2	(11.76)	0	(0)	0	(0)
Edema - chest	1	(5.88)	0	(0)	0	(0)
Fatigue	1	(5.88)	0	(0)	0	(0)
Granulation tissue at forearm donor site	1	(5.88)	0	(0)	0	(0)
Hallucinations-delirium	1	(5.88)	0	(0)	0	(0)
Heartburn	1	(5.88)	0	(0)	0	(0)
Hemoptysis	1	(5.88)	1	(5.88)	0	(0)
Hiccups	1	(5.88)	0	(0)	0	(0)
Hyperglycemia	2	(11.76)	0	(0)	0	(0)
Hypertension	2	(11.76)	0	(0)	1	(5.88)
Electrolytes (hypo): Ca+, K, mg, Na	7	(35.29)	0	(0)	0	(0)
Hypotension	3	(17.65)	0	(0)	0	(0)
Hypoxia	2	(11.76)	2	(11.76)	0	(0)
Increased sputum	1	(5.88)	0	(0)	0	(0)
Insomnia	1	(5.88)	0	(0)	0	(0)
Left shoulder neuropathy (nerve pain)	1	(5.88)	0	(0)	0	(0)
Leukocytosis	2	(11.76)	0	(0)	0	(0)
Low platelet count	1	(5.88)	0	(0)	0	(0)
Lymphedema	1	(5.88)	0	(0)	0	(0)
MRSA infection	1	(5.88)	0	(0)	0	(0)
Neck pain	1	(5.88)	0	(0)	0	(0)
Non-ST elevated myocardial infarction	1	(5.88)	0	(0)	1	(5.88)
Oropharyngeal dysphagia	1	(5.88)	0	(0)	0	(0)
Pain: surgical, neck, sacral, oral, hand, throat	8	(47.06)	0	(0)	0	(0)
Post op fever, no infection	1	(5.88)	0	(0)	0	(0)
Post op respiratory failure	1	(5.88)	1	(5.88)	0	(0)
Rash per Ancef	1	(5.88)	0	(0)	0	(0)
Sepsis	1	(5.88)	0	(0)	1	(5.88)
Shortness of breath	2	(11.76)	0	(0)	0	(0)
Superficial thrombosis	1	(5.88)	0	(0)	0	(0)

Thick secretions	1	(5.88)	0	(0)	0	(0)
Upper respiratory infection	1	(5.88)	0	(0)	0	(0)
Urinary tract infection	1	(5.88)	0	(0)	0	(0)
Vomiting	2	(11.76)	0	(0)	0	(0)
Weight loss	4	(23.5)	0	(0)	0	(0)
Wound breakdown	1	(5.88)	0	(0)	0	(0)

Supplementary Table 4a. Overall survival (OS) and disease-free survival (DFS) at different timepoints

	0-year	0.5-year	1-year	1.5- year	2- year	2.5- year	3-year
OS (%)	100 (100,100)	100 (100,100)	94 (84,100)	94 (84,100)	88 (74,100)	82 (65,100)	82 (65,100)
DFS (%)	100 (100,100)	82 (66,100)	76 (59,100)	71 (52,96)	71 (52,96)	71 (52,96)	71 (52,96)

Supplementary Table 4b. Actual number of patients at risk based on Gebski et al.[(*)]

	OS			DFS		
Time (months)	survival estimate	Actual # at risk	% information available	survival estimate	Actual # at risk	% information available
0	1	17		1	17	
6	1	17		0.82	15	100
12	0.94	16	100	0.76	13	100
18	0.94	16	100	0.71	12	100
24	0.88	15	100	0.71	12	100
30	0.82	13	97.3	0.71	11	100
36	0.82	8	97.3	0.71	6	100
42	0.82	5	97.3	0.71	3	100
48	0.82	1	97.3	0.71	1	100
54	0.82	1	97.3	0.71	1	100
60	0.82	1	97.3	0.71	1	100

Supplementary Table 5. IHC antibodies used in the study

Panel 1:

Antigen Retrieval	Antibody	Dilution	Incubation	TSA-Opal (PerkinElmer)	Dilution	Incubation
pH9	FOXP3 (Abcam 236A/E7)	1:100	45 min	540	1:150	10 min
pH6	PD-L1 (Cell Signaling E1L3N)	1:250	30 min	520	1:150	10 min
pH6	CD8 (Abcam SP16)	1:50	45min	570	1:150	10 min
pH6	CD3 (Abcam SP7)	1:50	45min	620	1:150	10 min
pH6	CD163 (Roche MRQ-26)	Pre-diluted	45min	650	1:150	10 min
pH6	CK (Dako AE1/AE3)	1:100	45min	690	1:100	10 min

Panel 2:

Antigen Retrieval	Antibody	Dilution	Incubation	TSA-Opal (PerkinElmer)	Dilution	Incubation
pH6	CD103 (Abcam EPR4166(2))	1:2000	30 min	690	1:150	10 min
pH6	CD8 (Abcam SP16)	1:50	45min	570	1:150	10 min
pH6	CD3 (Abcam SP7)	1:50	45min	620	1:150	10 min
pH6	Ki-67 (Cell Signaling D2H10)	1:50	30min	520	1:150	10 min
