# Supplementary material for: Neoadjuvant anti-OX40 (MEDI6469) therapy in patients with head and neck squamous cell carcinoma activates and expands antigen-specific tumor-infiltrating T cells
Source: Nat Commun. 2021 Feb 16;12:1047. doi: 10.1038/s41467-021-21383-1 (PMC7886909; doi:10.1038/s41467-021-21383-1)
Supplement: Supplementary file 3 — Description of Additional Supplementary Files [file 41467_2021_21383_MOESM3_ESM.pdf]

Description of Additional Supplementary Files

File Name: Supplementary Data 1
Description: Study protocol 14-042.

File Name: Supplementary Data 2
Description: Mutated neoantigen predictions for HNOX04, HNOX07 and HNOX18.

File Name: Supplementary Data 3
Description: Supporting statistical document.
