# Supplementary material for: Neoadjuvant anti-OX40 (MEDI6469) therapy in patients with head and neck squamous cell carcinoma activates and expands antigen-specific tumor-infiltrating T cells
Source: Nat Commun. 2021 Feb 16;12:1047. doi: 10.1038/s41467-021-21383-1 (PMC7886909; doi:10.1038/s41467-021-21383-1)
Supplement: Supplementary file 4 — Supplementary Data 1 [file 41467_2021_21383_MOESM4_ESM.pdf]

Phase Ib Study of a Monoclonal Antibody to OX40 (MEDI6469) Administered Prior to Surgery for Head and Neck Cancer

Version Date: 21 FEB 2017

Coordinating Center:
Providence Portland Medical Center
Earle A. Chiles Research Institute
Robert W. Franz Cancer Research Center
4805 NE Glisan St. Room 2N35
Portland, OR 97213
503-215-6259

Principal Investigator:
R. Bryan Bell, MD, DDS, FACS
Medical Director, Providence Oral Head and Neck Cancer Program
Providence Cancer Center
The Earle A. Chiles Research Institute
4805 NE Glisan Street, Suite 6N60
Portland, OR 97213
503-963-2874
richard.bell@providence.org

Co-Principal Investigator:
Rom Leidner, MD
The Earle A. Chiles Research Institute/Providence Cancer Center
4805 NE Glisan Street, 2N87
Portland, OR 97213
503-215-5696
503-215-5695 (FAX)
rom.leidner@providence.org

Co-Principal Investigator:
Brendan D. Curti, MD
The Earle A. Chiles Research Institute
4805 NE Glisan Street, 2N87
Portland, OR 97213
503-215-5696
503-215-5695 (FAX)
brendan.curti@providence.org

Co-Investigators:
Marka Crittenden, MD
Bernard Fox, PhD
Andrew Weinberg, PhD
Tuan Bui, MD, DDS
Eric Dierks, MD, DMD
Allen Cheng, MD, DMD
Carlo Bifulco, MD PhD
Brenda Fisher, RN
George, Morris, RN

PROVIDENCE
Cancer Center

PROTOCOL SIGNATURE PAGE

Protocol Number: 14-042 Version 21 FEB 2017

Protocol Title: Phase Ib Study of a Monoclonal Antibody to OX40 (MEDI6469) Administered Prior to Surgery for Head and Neck Cancer

I have read this protocol and agree that it contains all necessary details for carrying out this study. I will conduct the study as outlined herein and will complete the study within the time designated, in accordance with all stipulations of the protocol and in accordance with Federal Regulations, Good Clinical Practices, and local regulatory requirements.

I will provide copies of the protocol and all pertinent information to all individuals responsible to me who assist in the conduct of this study. I will discuss this material with them to ensure that they are fully informed regarding the study agent(s) and the conduct of the study.

R. Bryan Bell, MD	______________________	____________
**Investigator Name (print)**	**Investigator Signature**	**Date**

TABLE OF CONTENTS

PROTOCOL SIGNATURE PAGE 1

1. Summary of Study Proposal 3

2. Background 3
- 2.1 Treatment of oral head and neck cancer 3
- 2.2 HPV-related and HPV-unrelated OHNSCC's are distinct clinical entities 4
- 2.3 Immunotherapy and the importance of a pre-existent inflammatory T-cell response 4
- 2.4 Immune biology of oral head and neck cancer 5
- 2.5 OX40 biology and preclinical tumor models 6
- 2.6 Initial MEDI6469 Phase I Trial 8
- 2.7 Peri-operative immune therapy 10
- 2.8 Rationale for Phase Ib Study Design and Treatment Plan 11

3. OBJECTIVES 13

4. PATIENT SELECTION 14
- 4.1 Inclusion Criteria 14
- 4.2 Exclusion Criteria 14
- 4.3 Inclusion of Minorities 15

5 REGISTRATION PROCEDURES 15
- 5.1 Registration and Cancellation Guidelines 15
- 5.2 Assignment of Study Numbers 15

6 STUDY DESIGN 15

7 STUDY CALENDAR 17

8. ADMINISTRATION OF STUDY TREATMENTS 18
- 8.1 MEDI6469 Therapy 18
- 8.2 Surgical Therapy: Head and neck cancer resection and reconstruction. 19

9. MEASUREMENT OF EFFECT 19
- 9.1 Immunologic Monitoring 19
- 9.2 Peri-operative Monitoring (see Table 5 for Grading system) 20
- 9.3 Clinical Outcomes and Surveillance Database 21

10. ADVERSE EVENTS (AE) REPORTING AND MONITORING 22
- 10.1 Common Terminology Criteria for Adverse Events (CTCAE) 22
- 10.2 Definitions 23
- 10.3 AE Assessment 23
- 10.4 Reporting 25
- 10.5 Protocol Modifications and Amendments 26
- 10.6 Record Retention 26
- 10.7 Quality Assurance Plan 26
- 10.8 Study Monitoring 26
- 10.9 Quality Assurance 27
- 10.10 Plans For Assuring Accuracy Of Data 27

11. STATISTICAL CONSIDERATIONS 27
- 11.1 Sample Size and Statistical Analysis 27
- 11.1. Patient Accrual 28
- 11.2. Early Stopping Rules 28

APPENDIX A: PERFORMANCE STATUS CRITERIA 29

APPENDIX B: Autoimmune Disease Questionnaire 30

References 31

1. Summary of Study Proposal

OX40 is a potent co-stimulatory pathway that when triggered can enhance T-cell memory, proliferation and anti-tumor activity. A mouse monoclonal antibody that is an agonist to human OX40 signaling (MEDI6469) was recently tested in a phase I clinical trial and was found to be well tolerated and enhanced both humoral and cellular immunity in patients with metastatic cancer. Previous investigations at our institution have demonstrated that OX40 expressing T cells are found in abundance in the tumors of patients with advanced stage oral head and neck squamous cell carcinoma (OHNSCC), making this high-risk population of patients a compelling group in which to initiate OX40-directed therapy. Surgery is the primary treatment for most patients with OHNSCC, however, the effect of preoperative immune stimulation with a T-cell directed antibody and specifically OX40-directed therapy administration prior to surgical resection has not been tested in humans. Furthermore, there are limitations in accessing sufficient tumor sample for phenotypic and functional immunologic studies in the metastatic setting. We designed this phase Ib clinical trial using MEDI6469 at various dose intervals prior to definitive surgical resection of patients with stage III and IV OHNSCC with the primary objective of determining the safety and feasibility of preoperative MEDI6469 administration. In addition, we will obtain tumor tissues and peripheral blood for exploratory immunologic end points including measurements of tumor infiltrating immune cell populations based on flow cytometry and immunohistochemistry as well as circulating immunological parameters that may correlate with changes induced by MEDI6469 administration.

2. Background

Antibodies (Abs), that target T cell surface proteins, have recently been shown to be effective agents with which to stimulate anti-tumor immune response in preclinical and clinical settings.[1-5] So called “checkpoint inhibitors” or antagonists, such as anti-CTLA-4 and anti-PD-1, block negative signals to the T cells, while the agonists like anti-4-1BB and MEDI6469, enhance T cell function by increasing costimulation.[6] A positive phase III clinical trial of anti-CTLA-4, which demonstrated enhanced survival in patients with metastatic melanoma, led to the recent FDA approval of this antibody and has renewed enthusiasm for immunotherapeutic approaches to cancer.[7] Abs directed to PD-1 or PD-1 ligand have produced complete and partial responses as well as durable stable disease in patients with cancer.[8.9] The strategy of blocking inhibitory T cell pathways has shown clinical activity and there is ample preclinical evidence that T-cell co-stimulation via 4-1BB[10,11] or OX40 can induce immune-mediated rejection of tumors.[12-15] Our group is interested in exploring further the immunological and clinical properties of OX40 agonists. OHNSCC has a relatively poor prognosis with few treatment options and is known for its immunosuppressive properties, making it an ideal malignancy in which to test this immune stimulatory agent.

2.1 Treatment of oral head and neck cancer

OHNSCC typically arises in the oral cavity, oropharynx, larynx or hypopharynx, and is the 6th leading cause of cancer by incidence worldwide. Approximately 600,000 new cases are diagnosed each year, and only 40-50% of these patients will survive more than 5 years. Cancers that originate in the oral cavity and oropharynx account for 80% of OHNSCC’s and are characterized by a high rate of regional tumor extension, cervical lymph node metastasis and frequent distant metastasis.[16] Tobacco and alcohol are the major risk factors for OHNSCC, which appear to have a synergistic effect. Recently, human papillomaviruses (HPV) has also been shown to be an important independent risk factor for the development of OHNSCC.[17]

The prognosis of OHNSCC is largely determined by the stage at presentation and HPV status. Early staged tumors are treated by surgery or radiotherapy alone and have a favorable prognosis. The mainstays of treatment for advanced tumors are surgery combined with postoperative radiotherapy or chemoradiation, or by using organ-preservation protocols with combined platinum based chemo-radiation and surgery for salvage. Recently the use of targeted drugs has entered the field, most notably the application of cetuximab (anti-EGFR antibody) combined with radiotherapy.[18] While it is generally recognized that over the past two decades the quality of life of OHNSCC patients has increased by using more advanced surgical[19] and radiotherapeutic[20]

techniques, these technological advances have not resulted in significant improvement in disease-free or overall survival (reviewed below).

2.2 HPV-related and HPV-unrelated OHNSCC's are distinct clinical entities

HPV-unrelated OHNSCC is a distinct clinical entity, which responds much less favorably than HPV-related carcinomas to conventional therapies.[21] Several series have reported 3-year overall survival rates of 40-50% for patients with local regionally advanced HPV-negative OHNSCC treated with radiation and chemotherapy as opposed to 80% for those with HPV-positive carcinomas treated with similar regimens.[22] A recent meta-analysis by O'Rorke et al., which included forty-two studies and 4,843 patients documented that HPV-positive OHNSCC patients had a 54% overall survival benefit compared to HPV-negative patients and also had significantly improved progression-free and disease-free survival.[23]

Thus, despite technological advances in surgery and radiation, the defined inclusion of platinum-based chemotherapy into treatment approaches, and the FDA-approval of EGFR-inhibitors for use against OHNSCC, the advanced stage HPV-negative patient with OHNSCC continues to have a particularly poor prognosis, with a 5-year survival rate as low as 24% in some subsites.[24] Furthermore, their clinical course is characterized by frequent loco-regional recurrences, distant metastases and second primary tumors, due to a low response rate to current therapeutic strategies. There are strong indications that an immune-related component is responsible for the difference in outcomes between HPV-related and HPV-unrelated OHNSCC.[25]

It is in this population of HPV-negative and positive, locoregionally advanced OHNSCC that we propose this phase Ib clinical trial.

2.3 Immunotherapy and the importance of a pre-existent inflammatory T-cell response

It has long been observed that some OHNSCC's generate a robust inflammatory cell infiltrate within the tumor, at its invasive margin, and as lymphoid islets adjacent to the tumor; and other OHNSCC's demonstrate a paucity of lymphocytes. Recent investigations into the microenvironment of several malignant tumor types have demonstrated that many tumors are, in fact, infiltrated by lymphocytes of various types and functions and that the number, type and location of the tumor infiltrating lymphocytes (TIL's) may have prognostic significance.[26]

The realization that TIL's can be predictive of biological behavior[27] has led to the development of a method of quantifying and qualifying the immune infiltrate called "immunoscore".[28] This potential new prognostic biomarker is based primarily on the density of cytotoxic (CD8) and memory (CD45R0) T cells (CD3/CD45R0, CD3/CD8 or CD8/CD45R0) located at the tumor's center and at its invasive margin. In seminal investigations by Galon et al,[29,] Mlecnik et al[30] and Pages et al,[31] 'immunoscore' has been shown to be more prognostic than conventional TNM staging in colon cancer. Furthermore, these studies demonstrate that patients with high immunoscore have increased disease-free and overall survival compared to patients with low immunoscore. Validation studies in other tumor sites are currently underway through a large international consortium.[32]

Preliminary investigations of an OHNSCC tumor bank at our institution suggest that many tumors---particularly HPV-negative cancers, such as those that predominate in the oral cavity and larynx---are characterized by a paucity of inflammatory cells and would be considered "immunoscore negative" (Figure 1).[33] Furthermore, in a study by Wansom et al,[34] increased levels of TIL were correlated with a good prognosis and this fact might be exploited in the future to affect treatment decisions. Our group is interested in exploring the immunoscore concept further, with the goal of developing an immunotherapy strategy for the effective treatment of patients with OHNSCC. We hypothesize that the highly immune suppressed microenvironment and pervasive systemic immune suppression that characterizes OHNSCC may be reversed by a strategy that can increase anti-tumor inflammation via OX40 agonists.

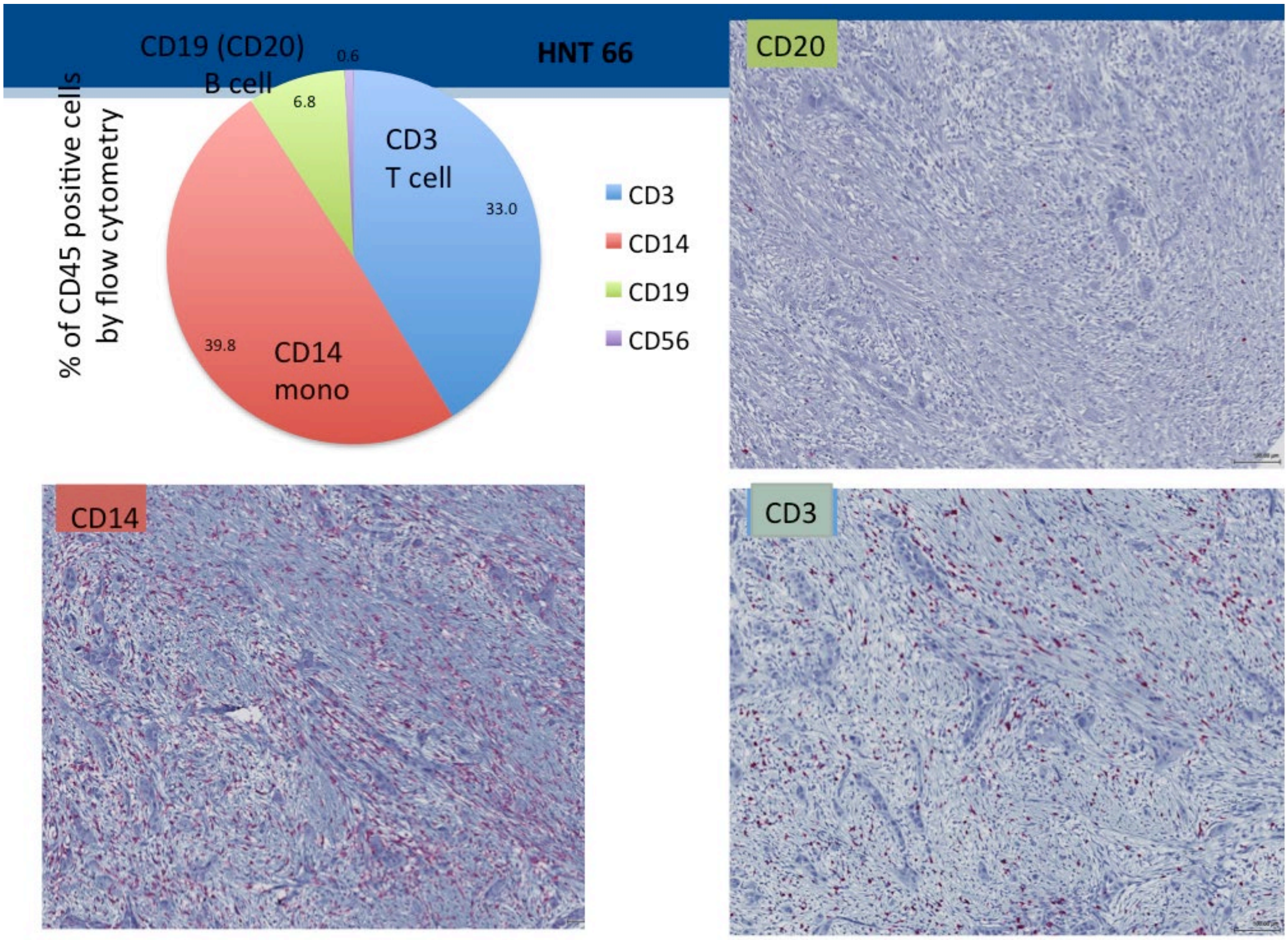

Figure 1. A preliminary study of 80 OHNSCC specimens (patient consented under master consent PHB IRB# 06-108A) has demonstrated the technical feasibility for the immunological monitoring of our proposed study and will serve as non-treated historical controls. This analysis has provided us with a detailed characterization of the TIL composition.

2.4 Immune biology of oral head and neck cancer

OHNSCC are notorious for their powerful immune suppression. Like other tumors, OHNSCC release suppressive factors that interfere with the differentiation and activation of dendritic and T cells, thus interfering with effective antitumor immunity.[35] High rates of apoptotic CD8+ T cell have been observed in tumors and in the circulation of patients with OHNSCC. Although the exact underlying mechanism remains to be established, apoptosis induction by circulating FasL-expressing microvesicles, by FasL+ and TRAIL+ tumor cells or by FasL or Granzyme-B expressing Tregs has been implicated.[36,37] Elevated levels of apoptosis sensitive CD8+CCR7- effector T cells and decreased levels of CD8+CCR7+ central memory T cells was reported in HNSCC patients with active disease, whereas patients with relatively high frequencies of CD8+CCR7+ T cells experienced significantly prolonged survival,[38] which is in line with earlier observations in colon cancer.[39] A transcriptional signature in OHNSCC consistent with an ongoing T cell response has similarly been associated with prolonged survival.[40] Accordingly, stage-dependent levels of T cell infiltration have been related to survival, with high frequencies of activated (CD8+) effector T cells correlating to improved survival and high levels of Tregs to late-stage disease and poor survival.[41,42] OHNSCC-derived suppressive factors can condition T cells and myeloid cells in such a way that they adopt suppressive features, converting them to Tregs and myeloid-derived suppressor cells (MDSC), respectively.[43] Chikamatsu et al[44] reported elevated levels of CD14+HLA-DR- MDSC in blood of OHNSCC patients and showed them to exert T cell-suppressive effects through PD-L1 and the release of TGFβ. In a recent publication MDSC isolated from blood, lymph nodes and tumors from patients with OHNSCC were shown to suppress T cell responses through arginase-1 activity.[45] STAT3 inhibition

interfered with this arginase-dependent suppression. STAT3 activation was also implicated in hampered activation of dendritic cells (DC), the most powerful antigen-presenting cell (APC) and as such vital to the generation of an effective antitumor T cell response. OHNSCC-derived supernatants blocked DC activation and interfered with their ability to attract lymphocytes; this effect was abrogated by STAT3 silencing in the OHNSCC cells prior to supernatant collection.[46] All the suppressive effects exerted by OHNSCC reduce an effective antitumor T cell response. Accumulating evidence points to the importance of an ongoing antitumor T cell response and tumor infiltration by active effector T cells *prior* to conventional therapy like surgery, chemotherapy or radiotherapy that correlates with clinical outcome.[47,48] We therefore propose a clinical strategy directed at promoting T-cell effector function and memory with the potential to induce anti-OHNSCC immunity and to lift suppressive conditions in the microenvironment.

2.5 OX40 biology and preclinical tumor models

OX40 is an important immune regulatory pathway that influences T-cell memory. OX40 is a member of the tumor necrosis factor (TNF) receptor family. The expression of OX40 is restricted to lymphoid tissue[49] and it is mainly expressed on activated CD4 and CD8 T cells.[50] After T-cell receptor (TCR) engagement by peptide antigen in the context of MHC class II, CD4 T cells exhibit peak expression of OX40 within 24-48 hours, which returns to baseline levels 120 hours later.[51] Effector CD4 T cells, which upregulate OX40 expression more rapidly than naïve T cells, express OX40 within 4 hours after antigen stimulation.[51] The transient expression of OX40 is observed both in vitro and in vivo.[52] $OX40^+$ T cells are found preferentially at sites of inflammation and not normally in the peripheral blood, although we have been able to identify OX40-stimulated T cells in the peripheral blood of patients who received an agonist OX40 antibody in our Phase I clinical trial (see section 2.6). In animal models for both autoimmunity and cancer, $OX40^+$ T cells were enriched for the recently stimulated auto- or tumor Ag-reactive T cells.[53,54,55] Therefore, OX40 represents a potential target to regulate the function of antigen-specific T-cell responses, even without prior knowledge of the specific antigens involved.[56]

OX40-expressing T cells are found in abundance in the primary tumors (Figure 2) and metastatic lymph nodes of OHNSCC patients (data not shown). OX40 expression was observed in significantly higher numbers of CD8 and conventional CD4 cells in the TIL of OHNSCC patients compared to the circulating T lymphocytes, as measured by flow cytometry of digested tissue versus PBL. Increased OX40 expression was particularly apparent in the $CD4^+$ $Foxp3^+$ $CD25^+$ $HLA\text{-}DR^+$ T regulatory population (Treg) present in the TIL and was irrespective of HPV status. The effect of an OX40 agonist on Treg may be very different than on non-Treg cells, and perhaps the proliferation and activity of non-Treg after MEDI6469 administration overcomes Treg inhibition, leading to tumor destruction. Observations from this clinical trial may shed light on this poorly understood area.

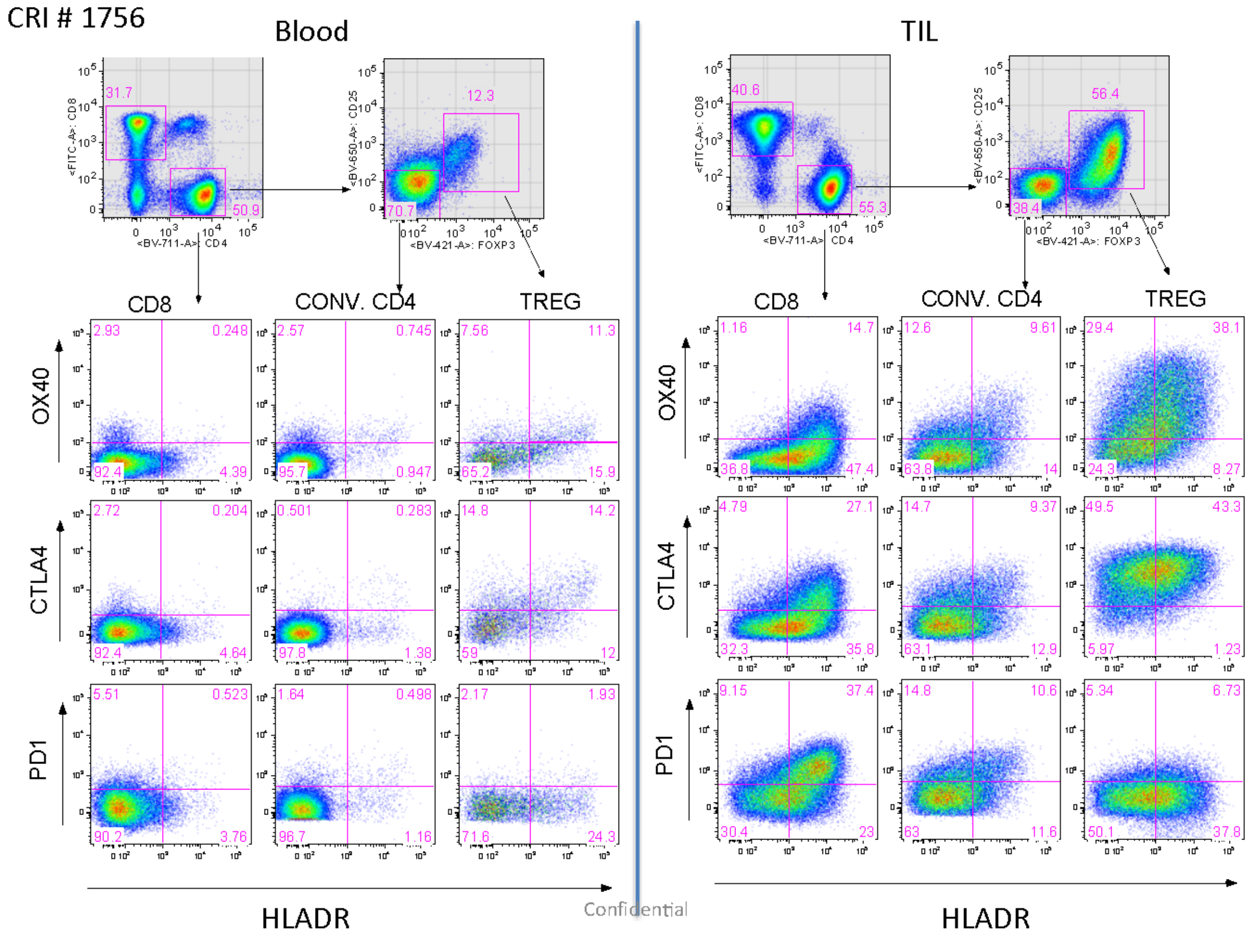

Confidential

Figure 2. OX40 expression in oral head and neck cancers by flow cytometry. Using a 10 color flow cytometry panel, we were able to characterize the subset of T cells present in the TIL and compare it to the patients blood, demonstrating increased OX40 expression on TIL.

Recently, our group and others have found that the immune stimulating properties of OX40 agonists can overcome some of the immunosuppressive properties of cancer. Injection of OX40 agonists leads to therapeutic responses in tumor-inoculated hosts in several preclinical cancer models including 4T-1 breast cancer, B16 melanoma, Lewis lung carcinoma and several chemically-induced sarcomas.[57-60] Changes in CD8 T cell number correlate with favorable therapeutic outcomes in several preclinical cancer models.

OX40 has costimulatory activity of similar potency to CD28 on an Ag-specific CD4+ T cell line in vitro[61] and was also shown to downregulate CTLA-4 protein expression on activated CD4 T cells[62]. While interaction of B7/CD28 is required for the optimal stimulation of naïve T cells.[63] OX40-specific costimulation appears to be an important signal for the stimulation and survival of effector T cells.[62,63] The control point for OX40-dependent stimulation of T cells during an immune response appears to be at the level of OX40 ligand (OX40L) expression.

The expression of OX40L is tightly regulated. When T cell activation via TCR engagement with peptide/MHC occurs in the absence of a strong adjuvant (as is the usual case for tumor-derived antigens), the local expression of OX40L is minimal. Therefore, in the absence of adjuvant, the antigen-stimulated T cells express OX40, but because OX40L expression on APC is limited, the majority of OX40+ T cells will never engage their natural ligand, and fail to achieve their maximum potential of proliferation, cytokine production, and survival.

While OX40 stimulation has been mainly associated with increased T cell survival, there is growing evidence that OX40-mediated co-stimulation can also greatly enhance both CD4 and CD8 effector T cell function.[64,65,66] In particular, CD4 T cells stimulated with antigen and MEDI6469 in vivo, recovered four days after priming from the draining lymph nodes, and restimulated with antigen ex vivo produced 10-fold greater levels of IFN-γ and IL-2 (on a per cell basis) compared to mice injected with antigen and rat Ig.[65] This observation was reproduced in a CD4 T cell tolerance model, where mice receiving an OX40 agonist were able to break tolerance and produce large amounts of IFN-γ compared to the control tolerant T cells [67,68] We have recently published that CD8 T cells stimulated with OX40 agonists greatly increased granzyme B production, which was associated with increased killing of antigen-specific targets, including tumor cells.[64] This finding was also confirmed in an adenoviral model, where OX40-deficient CD8 T cells had greatly reduced effector function.[66] The viral-specific OX40 knockout CD8 T cells showed reduced granzyme B levels and cell-mediated killing.

Another major cellular component within the tumor microenvironment (TME) are regulatory T Cells (Treg), which are defined by co-expression of CD4, Foxp3, and CD25. We have observed a high percentage of Treg within the TIL of patients with OHNSCC treated at our institution (Figures 2 & 3). Furthermore, we have demonstrated OX40 expression is significantly greater on Tregs in the TIL compared to the blood of OHNSCC patients. Recent reports have shown that OX40 agonists can inhibit both Treg function and the generation of TGF-b-inducible Treg(42).[69] Finally, a recent report described that the frequency of OX40 expressing cells was higher in the center of colorectal liver metastases than at the periphery.[70] Therefore, it appears that the MEDI6469 antibody can potentially have two targets: first, lymphocytes in the periphery (as observed in the initial phase I clinical trial); and second, OX40-expressing cells within the tumor. In preliminary studies at our institution we have observed a higher percentage of Treg cells from primary head and neck tumors when compared to the blood of the same patient. In general, the Treg cells isolated from the tumor have increased activation markers when compared to the blood. The percentage of Tregs or their activation status within the tumor could potentially be a predictive biomarker for patient outcome and may change following MEDI6469 administration to cancer patients.

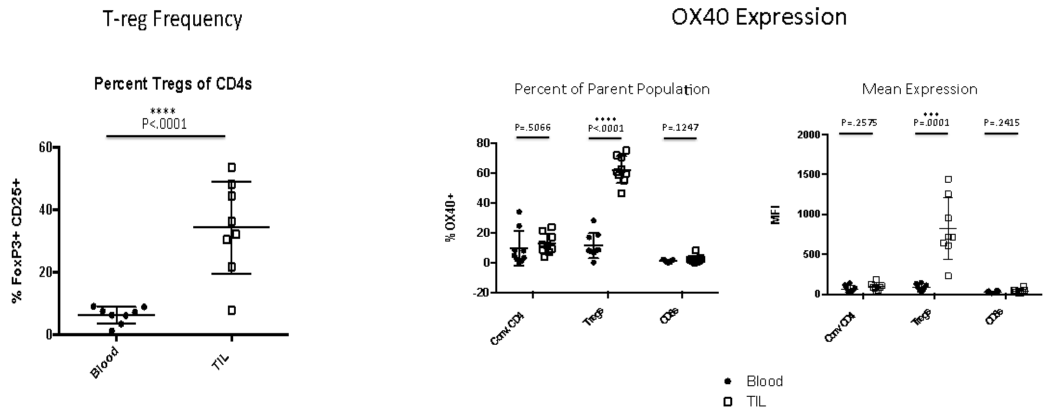

Figure 3. Treg frequency within TIL and OX40 expression on Treg in human patients with OHNSCC.

2.6 Initial MEDI6469 Phase I Trial

Patients with metastatic solid malignancies refractory to conventional therapy were enrolled in a phase 1 dose-escalation study using the 9B12 murine agonistic antihuman OX40 mAb, Trial ID#NCT01644968.[71] Consenting participants received a single cycle of MEDI6469 given intravenously (IV) on days 1, 3 and 5. The study was designed with three cohorts of ten patients each. Patients in cohort 1 received one cycle of 0.1 mg/kg, cohort 2, 0.4mg/kg and cohort 3, 2mg/kg of the mAb. The main objectives of the clinical trial were to determine the

maximum tolerated dose, toxicity, immunologic activity, and potential clinical activity following one cycle of MEDI6469.

Adverse events				
**Toxicity**	**Grade 1**	**Grade 2**	**Grade 3**	**Grade 4**
Lymphopenia	3	10	6	1
Fatigue	7	12		
Rash/Skin Changes	4	6		
Pruritis	5	1		
Fever/Chills	11	2		
Splenomegaly	7			
Arthralgias/Myalgias	5	5		
Nausea/Vomiting	4	3		
Increased AST, ALT or alkaline phosphatase	2	1		
Anemia	1	8		

Figure 4. Summary of maximum toxicities to MEDI6469 at all dose levels in Phase I MEDI6469 trial.

Toxicities were mild with grade 1-2 fatigue, transient lymphopenia and rash as the most common side effects (Figure 4). A maximum tolerated dose was not reached within the dose range examined. Of the 30 patients treated, no patients achieved a response using RECIST criteria, but 12 patients displayed regression of individual tumor deposits in lung, subcutaneous sites or lymph nodes and 6 others had stable metastatic disease for up to one year after MEDI6469 treatment (Figure 5). Immunologic changes were observed at all dose levels with significant proliferation of $CD4^+$ and $CD8^+$ central and effector memory T-cell populations. Proliferation of central and effector memory T cells was induced between 8 and 28 days after MEDI6469 administration.

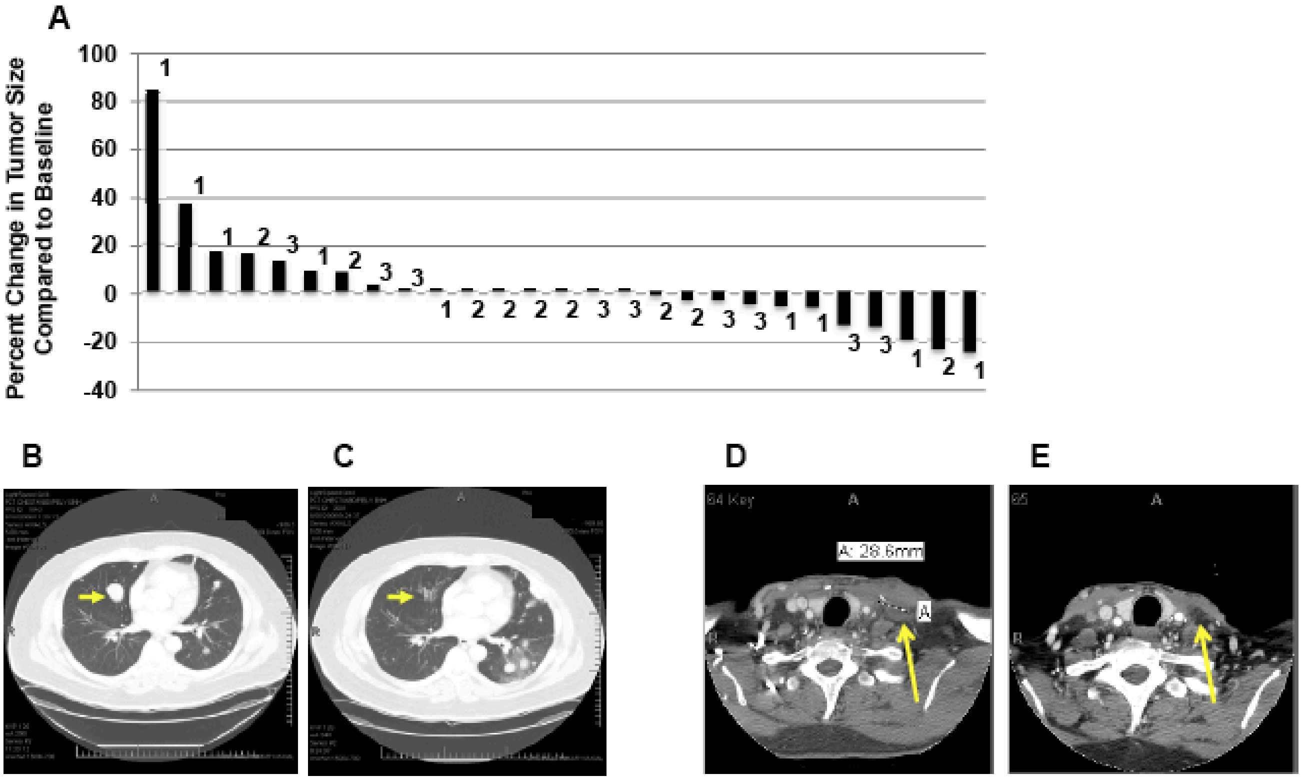

Figure 5. Clinical observations in phase I trial of MEDI6469**:** (A) Waterfall plot showing percent change in the sum of the diameters of tumor target lesions above or below baseline using RECIST criteria. The numbers on the graph indicate the cohort in which the patients were enrolled. Patients in cohort 1 received one cycle of 0.1mg/kg, cohort 2 0.4mg/kg and cohort 3 2mg/kg of MEDI6469. Patients 10, 18 and 23 did not have follow-up scans due to clinical progression and are PD. (B-C) Regression of a pulmonary nodule in a patient (Cohort 1) with metastatic melanoma, and progression of other nodules. Panel B, imaging before MEDI6469. Panel C, 5 months after MEDI6469 administration. (D-E) Shrinkage of a lymph node in a melanoma patient (cohort 3). Imaging in panel E was obtained 28 days after MEDI6469.

2.7 Peri-operative immune therapy

There is experimental evidence that MEDI6469 is safe and has no significant effect on wound healing. In mouse models, use of OX40 agonists preoperatively did not increase post-operative complications nor did it inhibit wound healing.[72] In this study, mice with an established sarcoma received MEDI6469 prior to surgery.

Animals that received MEDI6469 had a lower tumor recurrence and there was no wound healing delay observed (Figure 6).

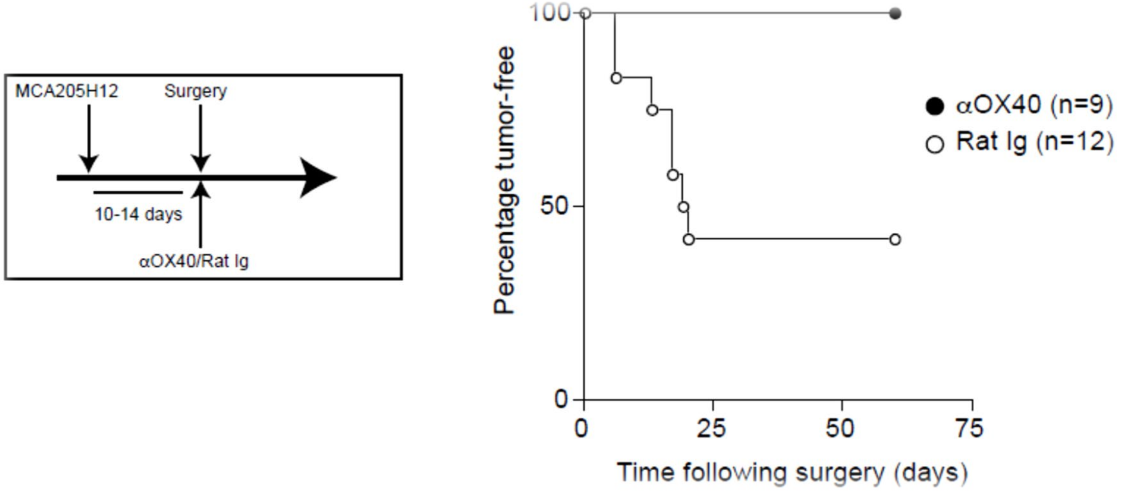

Figure 6. MCA205 tumors were established s.c. in the flank of C57BL/6 mice and were surgically removed when they reached 7 to 10 mm in diameter. At the time of the operation mice received a single injection of 250 mg of control Rat IgG or anti-OX40 antibody and followed for local tumor recurrence.

MEDI6469 has not yet been evaluated in the preoperative setting in humans. However, an antibody with a similar immunomodulatory function termed ipilimumab (anti-CTLA-4), has been evaluated in 12 patients with urothelial carcinoma of the bladder. Carthon et al,[73] found that ipilimumab in a pre-operative setting did not result in an increased frequency of post-operative complications. The post-operative complications that were observed were not attributed to the immunotherapy but comprised wound dehiscence as well as fistula in a patient with multiple prior abdominal surgeries and a urinary tract infection in 5 other patients. Importantly, investigators in the preoperative treatment model were able to gather data on intratumoral immune responses that identified a predictive biomarker of clinical response and overall survival when monitored in peripheral blood following treatment with ipilimumab. In resected tumor tissue obtained following ipilimumab treatment, $CD4^{+}ICOS^{hi}$ T cells were observed to be significantly increased in frequency as compared to archival tissue controls (p=0.004). This positively correlated with a 5 to 10-fold increase in circulating $CD4^{+}ICOS^{hi}$ T cells over pre-treatment baseline in peripheral blood at 7 weeks post-treatment (p=0.036). Analysis of cryopreserved peripheral blood samples from independent cohorts of metastatic melanoma patients, treated with or without ipilimumab, for circulating $CD4^{+}ICOS^{hi}$ T cells confirmed a significant increase in post-treatment circulating $CD4^{+}ICOS^{hi}$ T cells only in patients treated with ipilimumab (p=0.0012). Analysis of annotated clinical response and survival data demonstrated that a persistent increase in post-ipilimumab circulating $CD4^{+}ICOS^{hi}$ T cells, defined as at least a 2-fold increase in absolute number over baseline sustained at both weeks 7 and 12, was a statistically significant predictor of clinical benefit as assessed by response at 24 weeks (p=0.03) and overall survival (p=0.03, median OS not reached vs. 27 weeks).

2.8 Rationale for Phase Ib Study Design and Treatment Plan

Our previous research shows that 1) OX40 is a potent immune stimulating target for treatment that is highly expressed within the tumors of patients with head and neck squamous cell carcinoma; 2) MEDI6469 is well tolerated in humans and has an acceptable toxicity profile; 3) MEDI6469 favorably influences T cell responses to tumors. HPV-negative HNSCC is associated with a T-cell mediated immunosuppressive environment that is characterized by decreased levels of TIL and relatively large populations Tregs and other immunosuppressive factors. We hypothesize that administration of MEDI6469 will be safe in the preoperative setting and will result in local and systemic anti-tumor immune responses by inducing CD8-mediated effector T-cell proliferation and decreasing the suppressive activity of regulatory T cells; this amplified and directed immune response, prior to surgery, will enhance anti-tumor immunity and may control microscopic foci of residual disease.

The proposed trial is the first to evaluate the safety and feasibility of MEDI6469 in the preoperative setting. It is our expectation that there will not be increased risk of peri-operative complications based upon: 1) the mild toxicity observed in our phase I human trial;[71] 2) favorable wound healing observed on murine models of agonist OX40 administration prior to surgery;[72] and 3) successful wound healing observed in a trial of anti-CTLA 4 administered preoperatively to patients with urothelial carcinoma.[73]

The optimal dose and safety profile of MEDI6469 has been determined in the previous Phase I trial (see Section 1.4). There is a potential for MEDI6469 to have a negative effect on wound healing, because of the known role of OX40 as an immune stimulator, which may counteract the immune resolution phase of wound healing. Also, in the phase I study, the immunological effects of MEDI6469 lasted for at least 3 weeks. For these reasons, we will design the study to vary the timing of the MEDI6469 dose, starting with three weeks before surgery and decreasing the interval before surgery with each cohort, as long as no dose-limiting toxicity attributable to the medication is observed. The time course portion of the Phase Ib trial will be conducted as follows in Table 1:

Table 1: Timing of MEDI6469 administration prior to surgery

Dose	Cohort I	Cohort II	Cohort III
MEDI6469 0.4 mg/kg IV x 3 doses given on day 1, day 3-4, & day 5-6 of study	Surgery on day 19 +/- 2 of study	Surgery on day 12 +/- 2 of study	Surgery on day 8-9 of study

The time course has been chosen based on the activity and half-life of the mouse anti-human-OX40 antibody (2-3 days). The interval between MEDI6469 doses and resection will allow for determination of the effect of MEDI6469 on the TIL composition over time and will ensure patient safety in administering MEDI6469 preoperatively. After the time-course portion of the trial, an expansion cohort of up to 35 additional patients will be enrolled at the safe pre-operative dosing interval found to have the most promising immune response measured in peripheral blood and within tumors.

Modifying the composition of TILs by decreasing the proportion of Treg and increasing the proportion and potency of effector CD8 T cells may lead to an improved clinical outcome. In this study, we will compare the effect of MEDI6469 on the composition and immunologic phenotypes of lymphocyte subsets in TIL compared to historical controls. We will analyze the change in the proportion of $CD3^+$ T cells recovered from TIL following MEDI6469 therapy as well as the effect on CD4/CD8 ratio. The changes in $CD4^+$ and/or $CD8^+$ T cells proliferation will also be compared with the historical cohort. Overall Treg frequency and decrease in the highly suppressive phenotype ($CD4^+$ $Foxp3^+$ $CD25^+$ $HLA\text{-}DR^+$ Treg) will be measured and compared in resected tumors from historical cohorts; we will use this discrete cell population as our primary endpoint for the purposes of our sample size calculation (see section 11.1). Modification of the frequency of activated $CD8^+$ T cells as measured by the co-expression of HLA-DR and CD38 will also be evaluated. IHC studies will be performed on samples from patients enrolled in this trial to determine the distribution of T cells ($CD8^+$ T cells, conventional $CD4^+$ T cells and Treg cells) and to determine the effect of MEDI6469 on the localization of these different T cell populations. We will use the Immunoscore markers, CD8 and CD45RO to determine if anti-OX-40 treatment can modify the frequency of these cell populations that have been correlated with a positive clinical outcome in patients with metastatic colon cancer.

Table 2: Study Design

Phase 1b Pre-Surgical Interval: MEDI6469 prior to planned resection of head and neck malignancy (including but not limited to oral cavity, oropharynx, hypopharynx, larynx, salivary glands, thyroid and metastatic cutaneous).

Cohort I: 3 pts.

Surgery after 2 week interval

TIL analysis

→

Cohort II: 3 pts.

Surgery after 1 week interval

TIL analysis

→

Cohort III: 3 pts.

Surgery after 2-3 day interval

TIL analysis

→

Expansion Cohort: 35 pts.

Surgery at interval selected by time-course portion of the study (Cohort II was selected.)

TIL analysis

3. OBJECTIVES

The primary objectives of this phase Ib clinical trial are to examine the safety and feasibility of MEDI6469 administration prior to surgical resection of head and neck malignancy.

Exploratory objectives are as follows:

- Compare the effect of MEDI6469 administration on the composition and immunologic phenotypes of lymphocyte subsets isolated from peripheral blood (PBL), tumor-draining lymph node (uninvolved), primary tumor and metastatic lymph nodes.

- Compare the effect of MEDI6469 on the composition and immunologic phenotypes of lymphocyte subsets in primary tumor and metastatic lymph nodes compared to historical controls that did not receive MEDI6469.

- Monitor the clinical outcomes in patients receiving the MEDI6469 therapy compared to historical controls. Participants will provide consent at enrollment for inclusion in a deidentified, coded longitudinal database of clinical outcomes through five years surveillance post-treatment (see Section 9.3). This will include, but not be limited to: details of treatment, imaging, other testing, additional treatments, subsequent enrollment in clinical trials and results from clinical surveillance for recurrence as per current NCCN guidelines, which at a minimum presently include: PET +/- CT within 6 months of completion of treatment; physician visit for H&P at 3 month intervals years 1-2; 6 month intervals during years 3-5; and further imaging prompted by findings on history and exam.

4. PATIENT SELECTION

4.1 Inclusion Criteria

4.1.1 Patients planned for surgical resection via the Oral, Head and Neck cancer program at Providence Portland Medical Center.

4.1.2 Histologic or cytologic diagnosis of head and neck malignancy is acceptable. Patients with bone or cartilage invasion as well as distant metastasis are acceptable.

4.1.3 Eastern Cooperative Oncology Group (ECOG) performance status 0, 1, or 2 (Appendix A.)

4.1.4 Age 18 years or above.

4.1.5 Laboratory values (performed within 28 days prior to enrollment) as follows:

• WBC	≥2000/ L
• Hgb	>8g/dl (patients may be transfused to reach this level)
• Platelets	>75,000 cells/mm^3
• Serum creatinine	<1.5 X upper limit of laboratory normal
• Total bilirubin	<1.5 X upper limit of laboratory normal, unless due to Gilbert's disease
• AST (SGOT) and ALT (SGPT)	<2.5 X upper limit of laboratory normal
• Alkaline phosphatase	<2.5 X upper limit of laboratory normal
• HIV 1 and 2 antibody	Negative
• Hepatitis B surface antigen	Negative
• Hepatitis C antibody	Negative
• Procalcitonin	Normal range of lab assay

4.1.6 No clinical coagulopathy (INR <1.5, PT <16 seconds, PTT < 38 seconds).

4.1.7 Ability to give informed consent and comply with the protocol. Patients with a history of psychiatric illness must be judged able to understand the investigational nature of the study and the risks associated with the therapy.

4.1.8 No active gastrointestinal bleeding.

4.1.9 Anticipated lifespan greater than 12 weeks.

4.2 Exclusion Criteria

4.2.1 Active infection.

4.2.2 Active autoimmune disease including patients with Inflammatory Bowel Disease as determined by an autoimmune questionnaire (see appendix).

4.2.3 Previous treatment with mouse monoclonal antibodies

4.2.4 Need for chronic maintenance oral steroids > 5mg prednisone daily equivalent.

4.2.5 Any medical or psychiatric condition that in the opinion of the PI would preclude compliance with study procedures.

4.3 Inclusion of Minorities

Members of all ethnic groups are eligible for this trial; however, Oregon is 83.6% white based on 2011 census data and head and neck malignancy is primarily a disease of men. The expected distribution of men enrolled is based on our experience with other clinical trials at our Cancer Center. The anticipated study population is illustrated in Table 3, based on treating the maximum number of patients projected (also see statistics section).

Table 3.

	Race/Ethnicity					
Gender	White, not of Hispanic Origin	Black, not of Hispanic Origin	Hispanic	Asian or Pacific Islander	Unknown	Total
Male	28	2	4	2	0	36
Female	8	0	0	0	0	8
Total	36	2	4	2	0	44

5 REGISTRATION PROCEDURES

5.1 Registration and Cancellation Guidelines

To register a patient, the investigator will call the Data Management Office of the Robert W. Franz Cancer Research Center at (503) 215-2613 and speak to one of the Nurse Coordinators for the trial. The following information will be requested:

5.1.1 Investigator's name
5.1.2 Patient's Identification
5.1.3 Patient's name or initials and chart number
5.1.4 Patient's Social Security number
5.1.5 Eligibility Verification

Patients must meet all of the eligibility requirements and undergo all pre-study procedures.

If a patient enrolls in the study, but does not receive study therapy, the patient’s enrollment may be canceled. Reasons for cancellation will be documented in writing. Any patient whose enrollment was canceled before receiving study therapy will be replaced.

5.2 Assignment of Study Numbers

Study Numbers will be assigned at enrollment based on order of enrollment and initials. For example:

- MH is the 4th patient enrolled to study. Study Number is 4-MH
- BA is 9th patient enrolled to study. Study Number is 9-BA

All case report forms, study reports, and laboratory samples for research tests, including immune parameters or pharmacokinetics, will be labeled with the full patient Study Number.

6 STUDY DESIGN

Eligible patients will be registered (see Section 4) and assigned to a cohort:

Cohort 1 MEDI6469 0.4 mg/kg IV for 3 doses on day 1, 3 day 3-4, & day 5-6; Surgery on day 19 +/- 2
Cohort 2 MEDI6469 0.4 mg/kg IV for 3 doses on day 1, 3 day 3-4, & day 5-6; Surgery on day 12 +/- 2
Cohort 3 MEDI6469 0.4mg/kg IV for 3 doses on day 1, 3 day 3-4, & day 5-6; Surgery 2-3 days later

The rules for the time-course portion of the study are described in Table 4. Dose limiting toxicity (DLT) is defined as any grade greater than or equal to 3 non-hematologic toxicity (except hypothyroidism or vitiligo) that in the opinion of the investigator is considered at least possibly related to MEDI6469. Grade 3 or 4 hematologic toxicities that take longer than 10 days to resolve will be considered DLTs. Surgical complications (SC) will be recorded and graded using the Clavien-Dindo classification of surgical complications, which is described in greater detail in Section 9.2 (see Table 5). Timing interval reductions in individual patients will not be permitted. Standard supportive medications including anti-emetics, and pain medications will be offered during treatment.

For the time-course portion of the study, patients will be observed through post-operative day 14. Surgical complications (SC) would typically be observed within the first 2 weeks following surgery. Initially, one patient will be enrolled to each of the three time interval cohorts and observed through post-operative day 14. If no DLT are observed for the first patient in a given cohort, two additional patients may be enrolled to fill the cohort. If a DLT is observed for the first patient in a given cohort, only one additional patient at a time may be enrolled. If one of three total patients in a given cohort experiences a DLT, the cohort is expanded to six total patients, and three additional patients may be enrolled, (this need not be one at a time). Once two patients in a given cohort experience DLT, the cohort will be closed.

Table 4. Reduction in Interval between MEDI6469 and surgery

No. of Patients with DLT in a Given Cohort	Enter one patient to each of the three time interval cohorts and observe through post-operative day 14 for DLT.
**0 out of 1 patients:**<br>who undergo surgery	Enter 2 more patients to fill the current cohort.<br>• If 0 of 3 total patients experience DLT, cohort is complete<br>• If 1 out of 3 total patients suffer DLT, enroll 3 additional patients to complete cohort (need not be one at a time) ..<br>• Once 2 or more patients in a cohort suffer DLT, the current interval is declared closed.
**1 out of 1 patients:**<br>who undergo surgery	Enter 1 additional patient at a time to the current cohort.<br>• If 1 out of 3 total patients suffer DLT, enroll 3 additional patients to complete cohort.<br>• Once 2 or more patients in a cohort suffer DLT, the current interval is declared closed.

7 STUDY CALENDAR

Baseline evaluations, screening studies and labs within 28 days of the first day of treatment (CBC, CMP, PT, PTT, Procalcitonin, HBV SAg, HCV Ab, HIV 1 & 2 Ab), with the exception of pregnancy testing for pre-menopausal females (b-HCG), required on the day treatment starts.

Study Day	Screen Day -28	Day 1	Day 3-4	Day 5-6	Day 12 (+/- 2 days)	Day 34 (±3d)	Day 55 (±7d)	Follow up[10]
Informed Consent	X							
Demographics	X							
Fresh tumor biopsy[3]	X							
MEDI6469[1]		X	X	X				
Autoimmune tool[2]	X	X		X		X	X	
Surgery tissue[3]					X			
CT neck c+[4]	X				X			
Tumor Measurement	X				X			
Medical history	X	X			X	X	X	X
Physical exam	X	X			X	X	X	X
Vital Signs,	X	X			X	X	X	X
Weight/Height	X	X			X	X	X	X
Adverse Events[6]	X	X			X	X	X	
Performance status[7]	X	X			X	X	X	
CBC w/ Diff	X	X			X	X	X	
Standard CMP	X				X	X	X	
Immune Parameters[8]		X			X	X	X	
Hep B & C, HIV 1 & 2	X							
PT/PTT	X							
Procalcitonin	X							
CT chest to liver[11]	X							
b-HCG[9]		X						

1. MEDI6469 at 0.4 mg/kg IV over 60 minutes using in-line filter for 3 doses 48-96 hours apart.
2. Research RN to perform autoimmune disease assessment using assessment tool (see appendix B).
3. Fresh tissue and 3 Heparin Green 10ml tubes required prior to first dose of MEDI6469 and during surgical resection
4. Pre-op repeat CT neck c+ to assess changes in tumor volume for surgical planning purposes, per treating physician
5. Pre-op physical exam by physician will include objective measurement of primary tumor size based upon tattoo markings.
6. See Section 9.2 and Section 10 of protocol for adverse events grading.
7. See Appendix A for performance status definitions.
8. Collection of 50mL total whole blood in heparinized tubes (green top). Immune parameters are mandatory on day 1, pre-op on day of surgery, day 12 +/- 2 days, day 34 +/- 3d and day 55 +/- 7d. In addition, every effort should be made at surgery to obtain a tissue specimen of clinically uninvolved lymph node from the tumor draining basin for immune parameters.
9. Pregnancy testing with b-HCG is mandatory on day 1 of treatment for pre-menopausal females only
10. Follow-up after day 55 =/- 7d (the completion of the study) will be dictated by response and clinical standard of care. Additional treatment as deemed medically appropriate, such as risk-adapted adjuvant radiation +/- chemotherapy will be offered to patients. Following completion of primary treatment, clinical surveillance for recurrence will be performed as per current NCCN guidelines, which at a minimum presently include: PET +/- CT within 6 months of completion of treatment; physician visit for H&P at 3 month intervals years 1-2; 6 month intervals during years 3-5; and further imaging prompted by findings on history and exam. In the event of disease progression or recurrence, salvage therapy or subsequent clinical trials may be offered. A clinical outcomes and surveillance database, as described in Section 9.3, will be maintained by PI and delegated study coordinator, for recording events during follow-up for the five year interval post primary treatment.
11. PET/CT acceptable in place of CT alone.

8. ADMINISTRATION OF STUDY TREATMENTS

8.1 MEDI6469 Therapy

Manufacturing practices, stability, and composition of the study drug are contained in the investigators' brochure.

TOXICITY: Most toxicities have been grade 1 or 2. HAMA has been induced in 30/30 patients and generally appears by day 15 after MEDI6469. Fatigue and transient lymphopenia have been the most frequent toxicities observed. Erythematous skin rash lasting for 72 hours has been observed in 4/30 patients.

HOW SUPPLIED: MEDI6469 lyophilized Drug Product is supplied as a vialed lyophilized powder containing 50 mg (nominal) MEDI6469. When reconstituted with 1.2 mL of WFI, the solution contains 50 mg/mL MEDI6469, 10 mM sodium phosphate, 250 mM sucrose, 0.02% (w/v) polysorbate 80, at pH 7.5 (nominal 1 mL fill).

MEDI6469 Drug Product presentations do not contain preservatives and any unused portion must be discarded. Vials containing MEDI6469 may be gently inverted for mixing, but should not be shaken.

STORAGE: Must be stored at a temperature from 2 to 8° C. All clinical supplies will be stored in locked pharmacy facilities, in freezers with temperature monitoring. The Clinical Research staff and the research pharmacist at Providence Portland Medical Center review the monitor log.

PREPARATION/ADMINISTRATION: Lyophilized MEDI6469 Drug Product requires reconstitution prior to use. The reconstitution should be performed with 1.2 mL sterile WFI for each vial with the liquid added gently to the side of the vial to minimize product foaming. Commercially available sterile WFI will be supplied by the sites. The vial should be gently rotated or swirled for 5 minutes or until dissolution is complete. The vial should not be shaken or vigorously agitated. Reconstituted MEDI6469 should stand undisturbed at room temperature for a minimum of 5 minutes or until the solution clarifies. The reconstituted solution should appear clear or slightly opalescent. A thin layer of bubbles on the liquid surface is considered normal.

Preparation of investigational product MEDI6469 and preparation of the IV bag are to be performed aseptically. Total in-use storage time from reconstitution of MEDI6469 to the start of administration should not exceed 4 hours at room temperature or 24 hours at 2°C to 8°C (36°F to 46°F). If in-use storage time exceeds these limits, a new dose must be prepared from new vials.

Prepare a 50 mL 0.9% saline IV bag for dosing by withdrawing a volume of saline equal to the calculated dose volume from the IV bag.

Using aseptic handling, withdraw the contents of the vials into an appropriate sized syringe until the desired dose volume or the maximum capacity of the syringe is reached.

Add the contents to the selected 50 mL 0.9% (w/v) saline IV bag for all doses. Mix the bag by gentle inversion. Following preparation of the dose, the entire contents of the IV bag should be administered as an IV infusion using a 0.22 or 0.2-μm in-line filter. Flush the IV line with a volume of 0.9% (w/v) normal saline equal to the priming volume of the infusion set used after the contents of the IV bag are fully administered, or complete the infusion according to institutional policy to ensure the full dose is administered and document if the line was not flushed.

No incompatibilities between MEDI6469 and plastics passing compatibility tests (ie, polyethylene, polyolefin, or polyvinylchloride with di-2-ethylhexyl phthalate [DEHP] bags) have been observed. All DEHP-containing or DEHP-free lines are acceptable. Lines should contain a 0.22 or 0.2 μm in-line filter. The in-line filter can be made of polyethersulfone (PES) or polyvinylidene fluoride (PVDF). Lines containing cellulose-based filters should not be used with MEDI6469 as these have not been tested.

8.2 Surgical Therapy: Head and neck cancer resection and reconstruction.

HNSCC generally presents in subsites that include the oral cavity, oropharynx, hypopharynx and larynx. Surgery with risk adapted adjuvant therapy remains the primary modality for oral cavity tumors of all stages, and is a treatment option for T1 or T2 tumors of the oropharynx, hypopharynx and larynx. While definitive chemoradiation therapy is the treatment option of choice for advanced stage larynx cancer, there is some evidence to suggest that patients with tumors that invade the thyroid cartilage may have better locoregional control with upfront surgery followed by risk adapted adjuvant therapy. Surgical resection generally involves en bloc resection of the primary tumor with 1cm tumor-free margins, combined with cervical lymphadenectomy to clear the nodal basins at risk for harboring lymph node metastasis. Patients with T3 or T4 disease will usually require reconstruction with microvascular free tissue transfer. At our institution, this generally involves fibular osteocutaneous free flaps, radial forearm fasciocutaneous free flaps, or anterolateral thigh flaps.[74] Our group recently investigated the effect of preoperative chemotherapy or chemoradiotherapy on surgical complications in head and neck cancer patients and found there to be no difference in wound healing or flap success rates when patients had received prior chemotherapy or chemoradiation.[75] Use of anti-inflammatory drugs peri-operatively, (such as Toradol or other NSAIDS), will be at the discretion of the treating physician. However, after review of routine surgical practice by the study PI's, the specific use of glucocorticoid steroids by the surgeon (such as prednisone, prednisolone or dexamethasone) will not be allowed pre-operatively, or intra-operatively prior to completion of tissue harvest, under the study. Post-operative use of glucocorticoid steroids is to be avoided if at all feasible, but should not compromise patient care.

9. MEASUREMENT OF EFFECT

9.1 Immunologic Monitoring

The main objectives of the monitoring will be to characterize the composition and phenotype of lymphocytes in the TIL, tumor draining lymph node and in the peripheral blood. The sites performing these studies will include the Earle A. Chiles Research Institute (Immune Monitoring and Molecular Pathology Laboratories), MedImmune or outside vendors selected by the Principle Investigator and research team.

Blood will be delivered to the laboratory soon after it is drawn. The same day, the EACRI Immune Monitoring Lab (IML) will perform flow cytometry with a panel of markers including CD3, CD4, CD8, CD28, Ki-67, CD25, CD39, PD-1, OX40, HLA-DR, CTLA-4 and FoxP3. T cell sub-populations of interest include Treg and effector cells. This polychromatic flow assay will be used to identify proliferating T cells. Similar to the preliminary study described above, we will also procure a portion of the resected tumors at the time of resection. In addition, a shave biopsy of a clinically uninvolved lymph node from the tumor-draining basin will be routinely submitted, according to an IRB approved tissue procurement protocol which we have established during a recently completed study of dendritic cell subsets in OHNSCC tumor-draining lymph nodes. A laboratory representative will obtain the specimens in the operating room within 30 minutes of tumor extraction. The tumor will be digested to a single cell suspension, and will be incubated with antibodies in order to perform flow cytometry on TIL and tumor-draining lymph node. The panel of markers will be similar to those evaluated in the peripheral blood samples. In particular, we will examine the TIL for the presence of highly suppressive Treg and of the CD8 activated effector T cells. We will also look in the blood for the OX40 "immunologic signature," defined as the proliferation of memory and effector T cells, without proliferation of Treg observed in the Phase I clinical trial. Finally, paraffin-embedded tissue blocks will be obtained for immunohistological analysis of the resected tumors and the core biopsy specimens obtained intra-operatively prior to radiofrequency ablation. Antibodies anti CD3, CD45RO, CD8, CD4 and Foxp3 will be used to determine the localization of different lymphocytes in the tumor and in the normal tissue. This analysis will allow us to better characterize the distribution of different lymphocytes in patients that did not receive MEDI6469 (historical cohort) and compare it to patients treated with the MEDI6469.

9.1.1 Immunoscore and immune profiling

Tumor biomarker studies of immune cells infiltrating the tumor or tumor margin suggest that patients with a pre-existing immune response have a better outcome than patients lacking this activated “Immunoscore” or “Immune gene” signature.[28-31] While these data have yet to be validated and await confirmation, a large number of reports in a wide spectrum of solid cancer histologies suggest an important pattern that is consistent with the “immunoediting” hypothesis.[76] This underscores the importance of performing immunological biomarker studies on initial cancer diagnosis biopsies. Additionally, it raises the important question whether OX40-induced anti-cancer immune responses can improve the immunoscore or gene signature. The sites performing these studies will include the Earle A. Chiles Research Institute, MedImmune or outside vendors selected by the Principle Investigator and research team.

Our institute has created a head and neck cancer tumor bank, which currently consists of more than 100 cryopreserved tumor specimens, >250 paraffin embedded tissues, 9 tumor cell lines and 32 TIL cultures from OHNSCC patients that will be available to assess whether the tumor immune infiltrate can be of prognostic value for OHNSCC. We will use tissue-microarrays (TMA) generated from tissue of all stages (I-IV) of OHNSCC (oral cavity, larynx, pharynx). TMA from the MEDI6469 study group will be used for a test cohort. Another group of 200-250 patient samples will also be used to make TMA that can serve as a validation cohort. Both HPV-negative and HPV–positive tumors will be included on these arrays. Whereas 4-6 color analyses might be available at the start of this project, we currently propose 3-color analysis identifying two immune markers in combination with a nuclear stain (DAPI). Slides will be stained using a Benchmark XT autostainer, imaged using a Leica SCN400 F Brightfield and Fluorescence Slide Scanner and analyzed using the Definiens analysis software[83] and methods standardized for a global immunoscore project. We are currently already optimizing this technique at the EACRI for other tumor types such as NSCLC and melanoma. We will be able to adjust protocols and panels based on those optimization studies. In addition to the tumor immune infiltrate, we will determine the peripheral immune state by flow cytometric profiling of peripheral blood and LN and perform an assessment of the immune response following MEDI6469 in PBMC and LN samples from OHNSCC patients (“peripheral immunoscore”). To further ascertain why certain samples are predominantly negative for immune infiltrates, or show a highly suppressive infiltrate, we may explore additional platforms, such as in situ hybridization (ISH), to detect message for cytokines, chemokines and suppressive molecules such as IDO and Arginase. These assays are currently under development at the EACRI in Dr. Bernard Fox’s laboratory. Based on the most valuable and scientifically most interesting correlations between the marker sets analyzed in these samples, we will determine which immune parameters will ultimately be included in subsequent/future studies addressing a hypothesis that a tumor immune infiltrate that is predominantly skewed to an effector phenotype with minimal suppressive/regulatory components will correlate with improved progression-free and overall survival in OHNSCC patients, whereas lack of an effector infiltrate and/or a predominant regulatory/suppressive infiltrate will correlate with reduced progression-free and overall survival.

9.2 Peri-operative Monitoring (see Table 5 for Grading system)

Once the patients are enrolled in the study, the primary tumor objectively measured at scheduled time points specified in the study calendar, **section 7**. A study physician will examine patients weekly during the interval prior to surgery, starting on day 1 with first MEDI6469 dose. Post-operative monitoring for complications, and long-term tumor-specific outcomes will be continued at scheduled intervals as indicated in the study calendar through day 55 +/- 7 days, and thereafter per institutional standard of care, (e.g., patients will be seen and examined by a physician every 3 months, and will undergo routine lab tests, as well as imaging studies to evaluate for tumor recurrence per the primary physician). Adjuvant radiation therapy and chemotherapy regimens are not limited by inclusion in this study, and will be determined by the oncology treatment team.

There are currently no standard guidelines or criteria by which to report complications in the area of head and neck surgery. The lack of consensus on how to define and grade adverse postoperative events has greatly hampered the evaluation of surgical procedures. A new classification of complications, called the Clavien-Dindo classification, was first proposed for use in urological surgery in 1992, has since been modified, and is now widely used by a number of surgical subspecialists to describe surgical complications.[77] It is based on the type

of therapy needed to correct the complication, as opposed to describing “minor and major complications, which is subjective and prone to bias and inconsistency. The Clavien-Dindo classification was recently reviewed and validated as a tool that is applicable for a wide range of surgical subspecialties. Each patient enrolled in the current study will receive a grade based upon any deviation from the normal postoperative course, as defined by the Clavien-Dindo classification system in **Table 5**. Patients that do not experience a complication will receive a grade of “0”.

9.3 Clinical Outcomes and Surveillance Database

A coded, de-identified clinical database will be maintained by the study PI or study coordinator as delegated by the study PI. The database and codes key will be housed on a password and encryption protected network drive, only accessible to the study PI and a delegated research coordinator. Participants will provide consent for inclusion in the longitudinal study database at enrollment. Clinical data will be collected from time of enrollment to a minimum of five years clinical surveillance post completion of treatment, or death or loss to follow-up. Such data will include but not be limited to: date and age at diagnosis, diagnostic work-up, staging and ancillary/molecular studies, tobacco & alcohol history, family history of cancers, comorbid conditions, medications used, surgical procedures, treatment details including radiation and chemotherapy, tissue accession numbers, imaging results, enrollment in any subsequent clinical trials. Clinical surveillance for recurrence will be performed as per current NCCN guidelines and results recorded in the database, which at a minimum presently include: PET +/- CT within 6 months of completion of treatment; physician visit for H&P at 3 month intervals years 1-2; 6 month intervals during years 3-5; and further imaging prompted by findings on history and exam.

Table 5.

CLAVIEN-DINDO GRADING SYSTEM FOR THE CLASSIFICATION OF SURGICAL COMPLICATIONS

Grades	Definition
Grade I:	Any deviation from the normal postoperative course without the need for pharmacological treatment or surgical, endoscopic and radiological interventions. Allowed therapeutic regimens are: drugs as antiemetics, antipyretics, analgetics, diuretics and electrolytes and physiotherapy. This grade also includes wound infections opened at the bedside.
Grade II:	Requiring pharmacological treatment with drugs other than such allowed for grade I complications. Blood transfusions and total parenteral nutrition are also included.
Grade III:	Requiring surgical, endoscopic or radiological intervention
Grade III-a:	Intervention not under general anesthesia
Grade III-b:	Intervention under general anesthesia
Grade IV:	Life-threatening complication (including CNS complications: brain haemorrhage, ischaemic stroke, subarachnoid bleeding, but excluding transient ischaemic attacks) requiring IC/ICU management.
Grade IV-a:	Single organ dysfunction (including dialysis)
Grade IV-b:	Multi-organ dysfunction
Grade V:	Death of a patient
Suffix 'd':	If the patients suffers from a complication at the time of discharge, the suffix "d" (for 'disability') is added to the respective grade of complication. This label indicates the need for a follow-up to fully evaluate the complication.

10. ADVERSE EVENTS (AE) REPORTING AND MONITORING

10.1 Common Terminology Criteria for Adverse Events (CTCAE)

This study will utilize the Common Terminology Criteria for Adverse Events (CTCAE) active version (v4.0). The CTCAE active version can be accessed from the CTEP home page (http://ctep.cancer.gov).

10.2 Definitions

Adverse event:
Any unfavorable and unintended sign (including an abnormal laboratory finding), symptom, or disease temporally associated with the use of a medical treatment or procedure regardless of whether it is considered related to the medical treatment or procedure (attribution of unrelated, unlikely, possible, probable, or definite). Medical conditions or diseases present before starting the investigational drug will be considered as treatment-related AEs if they worsen after starting study treatment.

Serious adverse event:
Any adverse drug experience occurring at any dose that results in any of the following outcomes: Death, a life-threatening adverse drug experience, inpatient hospitalization or prolongation of existing hospitalization, a persistent or significant disability/incapacity, or a congenital anomaly/birth defect. Important medical events that may not result in death, be life-threatening, or require hospitalization may be considered a serious adverse drug experience when, based upon appropriate medical judgment, they may jeopardize the patient or subject and may require medical or surgical intervention to prevent one of the outcomes listed in this definition.

Unexpected adverse event:
Any adverse drug experience, the specificity or severity of which is not consistent with the current investigator brochure; or, if an investigator brochure is not required or available, the specificity or severity of which is not consistent with the risk information described in the general investigational plan or elsewhere in the current application, as amended.

Disability:
A substantial disruption of a person's ability to conduct normal life functions.

Life-threatening adverse drug experience:
Any adverse drug experience that places the patient or subject, in the view of the investigator, at immediate risk of death from the reaction as it occurred, i.e., it does not include a reaction that, had it occurred in a more severe form, might have caused death.

Unanticipated Problem:
An unanticipated problem is an adverse event that is (i) unexpected; (ii) serious; and (iii) felt by the investigator to be possibly, probably, or definitely related to the research intervention. Only adverse events that meet this definition need be reported to the IRB.

For more information on the definition of an unanticipated problem and reporting requirement, consult the current PH&S AE Guidelines published on the IRB website.

10.3 AE Assessment

To assess AEs, first identify and grade the severity of the event using the CTCAE. Next, determine whether the event is expected or unexpected and if the event is related to the medical treatment or procedure. With this information, determine whether an adverse event should be reported as an expedited report and/or reported to the PH&S IRB.

10.3.1. Expected vs. Unexpected

- The determination of whether an AE is expected is based on the agent-specific information provided in Section 12 of this protocol.
- Unexpected AEs are those not listed in the agent-specific information provided in Section 12 of this protocol.

10.3.2. Assessment of Attribution

The following will be used to assess the relationship of an adverse event with the study agent:

Definite – Applies to those adverse events which, the Investigator feels are incontrovertibly related to study agent. An adverse event may be assigned an attribution of definitely related if or when (must have all of the following):

- It follows a reasonable temporal sequence from administration of the test drug.
- It could not be reasonably explained by the known characteristics of the subject's clinical state, environmental or toxic factors, or other modes of therapy administered to the subject.
- It disappears or decreases on cessation or reduction in dose with re-exposure to drug. (Note: this is not to be constructed as requiring re-exposure of the subject; however, the group of definitely related can only be used when a recurrence is observed.)
- It follows a known pattern of response to the test drug.

Probable – Applies to those adverse events for which, after careful consideration at the time they are evaluated, are felt with a high degree of certainty to be related to the test drug. An adverse event may be considered probably related if or when (must have three of the following):

- It follows a reasonable temporal sequence from administration of the test drug.
- It could not be reasonably explained by the known characteristics of the subject's clinical state, environmental or toxic factors, or other modes of therapy administered to the subject.
- It disappears or decreases on cessation or reduction in dose. There are important exceptions when an adverse event does not disappear upon discontinuation of the drug, yet drug-relatedness clearly exists (e.g. bone marrow depression, fixed drug eruptions, tardive dyskinesia).
- It follows a known pattern of response to the test drug.

Possible – Applies to those adverse events for which, after careful consideration at the time they are evaluated, a connection with the test drug administration appears unlikely but cannot be ruled out with certainty. An adverse event may be considered possibly related if or when (must have two of the following):

- It follows a reasonable temporal sequence from administration of the test drug.
- It could not readily have been produced by the subject's clinical stated, environmental or toxic factors, or other modes of therapy administered to the subject.
- It follows a known pattern of response to the test drug.

Unlikely – Applies to those adverse events for which, after careful consideration at the time they are evaluated, are judged to be unrelated to the test drug. An adverse event may be considered unlikely if or when (must have two of the following):

- It does not follow a reasonable temporal sequence from administration of the test drug.
- It could readily have been produced by the subject's clinical state, environmental or toxic factors, or other modes of therapy administered to the subject.
- It does not follow a known pattern of response to the test drug.
- It does not reappear or worsen when the drug is re-administered.

Unrelated – Applies to those adverse events, which after careful consideration, are clearly and incontrovertibly due to extraneous causes (disease, environment, etc.).

10.4 Reporting

10.4.1. PH&S IRB Reporting

An unanticipated event that is serious and definitely or probably caused by the study treatment (drugs or device) will be reported to the IRB in accordance with their guidelines and within their timelines.

**SAE Reporting Requirements**								
	**Grade 1**	**Grade 2**	**Grade 2**	**Grade 3**		**Grade 3**		**Grades 4 & 5**
	**Unexpected and Expected**	**Un-expected**	**Expected**	**Unexpected**		**Expected**		**Unexpected and Expected**
				**with Hospitali-zation**	**without Hospitali-zation**	**with Hospitali-zation**	**without Hospitali-zation**	
**Unrelated Unlikely**	Not Required	Not Required	Not Required	10 Calendar Days	Not Required	10 Calendar Days	Not Required	24-Hour; 5 Calendar Days
**Possible Probable Definite**	Not Required	10 Calendar Days	Not Required	24-Hour; 5 Calendar Days	24-Hour; 5 Calendar Days	10 Calendar Days	Not Required	24-Hour; 5 Calendar Days
**Note: SAE reporting is NOT REQUIRED for Grade 3 or 4 lymphopenia**								

10.4.2. Expedited Adverse Event Reporting Requirements

FDA:
The sponsor shall notify FDA using MedWatch 3500A (Mandatory Reporting Form) of: (A) Any adverse experience associated with the use of the drug that is both serious and unexpected; or (B) Any finding from tests in laboratory animals that suggests a significant risk for human subjects including reports of mutagenicity, teratogenicity, or carcinogenicity. Each notification shall be made as soon as possible and in no event later than fifteen (15) days after the sponsor's initial receipt of the information. Each written notification to FDA shall be transmitted to the FDA new drug review division in the Center for Biologics Evaluation and Research that has responsibility for review of the IND.

Telephone and facsimile transmission safety reports. The sponsor shall also notify FDA by telephone or by email transmission of any Grade 3-5 autoimmune events as well as any unexpected fatal or life-threatening experience associated with the use of the drug as soon as possible but in no event later than seven (7) days after the sponsor's initial receipt of the information. Each telephone call or facsimile transmission to FDA shall be transmitted to the FDA new drug review division in the Center for Biologics Evaluation and Research that has responsibility for review of the IND.

10.4.3. Data Reporting

Clinical data will be recorded on study-specific case report forms (CRFs). All forms must be legible and complete. Black ink must be used in completing these forms. All corrections must be noted with a single line strike though inaccurate entries and will be initialed and dated. All data entries to CRFs must be supported by a clinical source document. No direct entry of patient data to the CRF is permitted.

10.4.4. Continuing Review and Final Reports

An annual progress report (continuing review) will be submitted to the IRB and FDA (for IND) for the duration of the study. Continuing review reports to the FDA will comply with Title 21, Part 312.33 of the Code of Federal Regulations. All IRB continuing reviews and confirmation from the IRB will be forwarded to the FDA.

10.5 Protocol Modifications and Amendments

All modifications or amendments to the protocol or informed consent document must be approved by the Principal Investigator and submitted to the IRB for review and approval. All modifications and amendments will be documented with a new version number and date. All changes to the informed consent document will include the date of the revision on the form.

No changes will be implemented until IRB approval is obtained except when a potential threat to patient safety exists.

The IRB will be notified of any significant deviations from the approved protocol. Documentation of all IRB correspondence will be maintained in the central regulatory file according to section 10.5.

10.6 Record Retention

According to 21 CFR 312.62(c), the investigator shall retain required records for a period of 2 years following the date a marketing application is approved for the drug for the indication for which it is being investigated. If no application is to be filed or if the application is not approved for such indication, the investigator shall retain these records until 2 years after the investigation is discontinued (IND is withdrawn) and the FDA is notified.

The investigator must retain protocols, amendments, IRB/IBC approvals, copies of the Form FDA 1572, completed, signed, dated consent forms, patient source documents, case report forms, quality monitoring reports, drug accountability records and all documents of any nature regarding the study or patients enrolled. All records will be maintained under restricted access by the Clinical Trials Department at Providence Portland Medical Center while the study remains active. Records may be placed in long-term storage after the study is completed. The location of long-term storage will be secure and easily accessed for regulatory purposes.

10.7 Quality Assurance Plan

The Providence Health System Quality Assurance (QA) plan for cancer clinical trials comprises Standard Operating Procedures (SOPs) that require ongoing review of activities associated with all investigator-initiated trials including protocol compliance, accuracy of data and safety of participants.

10.8 Study Monitoring

Clinical research staff members who have completed specialized training in study monitoring procedures and human subjects protections perform study monitoring activities (Quality Control Reviews). Individuals who perform study monitoring activities do not report to Principal Investigators or research scientists and may not monitor studies for which they have direct responsibility.

Study monitoring activities are conducted regularly and include (but are not limited to) review and verification of the following:

- Eligibility
- Informed Consent process
- Adherence to protocol treatment plan
- Case Report Forms (CRFs)
- Source Documentation

- Adverse Events
- Regulatory Reporting

Results of study monitoring activities will be reported to applicable study personnel, the Clinical Trials Manager and Quality Assurance.

10.9 Quality Assurance

Quality Assurance (QA) personnel review study monitoring reports and if necessary, determine follow-up actions to resolve significant findings. QA has the authority to request immediate corrective action if significant patient safety issues are identified.

QA will track and trend results from study monitoring reports as well as associated corrective and preventive actions. A QA summary report will be provided to the IRB at the time of continuing review.

QA personnel do not have a direct reporting relationship to the Principal Investigator and are not responsible for enrollment or coordination of care for study participants.

10.10 Plans For Assuring Accuracy Of Data

All case report forms will undergo quality assurance review. All quality assurance reviews will include verification of the accuracy and integrity of data entered to case report forms. Incorrect data will be identified and corrected. The existence of adequate source documents for all data will be verified. A staff person not associated with patient care coordination, data completion or submission will review all annual reports.

11. STATISTICAL CONSIDERATIONS

11.1 Sample Size and Statistical Analysis

The primary objective of this trial is to examine the safety and feasibility of definitive surgical resection following MEDI6469 administration for locoregionally advanced OHNSCC.

This study will start with a Phase Ib time-course design using a constant MEDI6469 dose based on the previous Phase I trial, given at varying time interval prior to planned surgical resection. This will be followed by an expansion cohort enrolling up to an additional 35 patients at the safe pre-operative dosing interval found to have the most promising immune response measured in peripheral blood and within tumors. The initial Phase I portion of the study will include at least 9 patients who undergo resection, using enrollment rules described in Section 6. The protocol may be amended to revise patient number based on clinical or immune response, and/or change in OX40 supply.

For exploratory objectives described in Section 3, a total of up to 38 patients will have received MEDI6469 at the immunologically most promising pre-surgical interval (3 + 35 expansion). Allowing for 15% sample attrition, in the case we are unable to acquire valid measurements from some samples, will provide a sample size of at least N=32 for the most immunologically promising pre-surgical schedule. Control samples from the established and growing institutional bioarchive described in Section 9.1.1 will be matched one-to-one with study patients based on gender, anatomic primary site, stage, HPV status and smoking status. Exploratory analyses of immunologic studies will include means, frequencies, correlations, chi-square tests, and t-tests. For 2-sided t-tests of tumor tissue, tissue at tumor margins, blood samples, and lymph node samples in the treatment samples and historical controls, a sample size of 32 per group provides greater than 80% power to detect a 1.5 fold difference in group means of cell counts, such as Tregs and effector cells and others described in Section 9.1, assuming a coefficient of variation of .55 as observed in the Tregs of the phase 1 trial previously completed. Log base 10 transformations of cell counts will be used if data are log normal.

11.1. Patient Accrual

Our surgeons perform over 100 OHNSCC resections annually, with roughly 50% HPV-positive and 50% HPV-negative cases. It is estimated that we can enroll two to three patients per month to this trial.

11.2. Early Stopping Rules

Early stopping of this trial will be based on unacceptable toxicity, defined as acute (within 90 days of the start of treatment) or late (more than 90 days from the start of treatment) grade 4 or 5 toxicity (per CTCAE, v.4.0). If a patient has more than one unacceptable toxicity, they will only be counted as one unacceptable toxicity for this analysis.

APPENDIX A: PERFORMANCE STATUS CRITERIA

**ECOG Performance Status Scale**		**Karnofsky Performance Scale**	
Grade	Descriptions	Percent	Description
0	Normal activity. Fully active, able to carry on all pre-disease performance without restriction.	100	Normal, no complaints, no evidence of disease.
		90	Able to carry on normal activity; minor signs or symptoms of disease.
1	Symptoms, but ambulatory. Restricted in physically strenuous activity, but ambulatory and able to carry out work of a light or sedentary nature (e.g., light housework, office work).	80	Normal activity with effort; some signs or symptoms of disease.
		70	Cares for self, unable to carry on normal activity or to do active work.
2	In bed <50% of the time. Ambulatory and capable of all self-care, but unable to carry out any work activities. Up and about more than 50% of waking hours.	60	Requires occasional assistance, but is able to care for most of his/her needs.
		50	Requires considerable assistance and frequent medical care.
3	In bed >50% of the time. Capable of only limited self-care, confined to bed or chair more than 50% of waking hours.	40	Disabled, requires special care and assistance.
		30	Severely disabled, hospitalization indicated. Death not imminent.
4	100% bedridden. Completely disabled. Cannot carry on any self-care. Totally confined to bed or chair.	20	Very sick, hospitalization indicated. Death not imminent.
		10	Moribund, fatal processes progressing rapidly.
5	Dead.	0	Dead.

APPENDIX B: Autoimmune Disease Questionnaire

Does the patient have any of the following conditions?	YES	NO
Graves' disease	☐	☐
Active Hashimoto's thyroiditis	☐	☐
Autoimmune polyglandular syndrome	☐	☐
Insulin-resistant diabetes mellitus	☐	☐
Immune-mediated infertility	☐	☐
Autoimmune Addison's disease	☐	☐
Pemphigus vulgaris	☐	☐
Pemphigus foliaceus	☐	☐
Dermatitis herpetiformis	☐	☐
Autoimmune alopecia	☐	☐
Active psoriasis	☐	☐
Psoriatic arthritis	☐	☐
Autoimmune hemolytic anemia	☐	☐
Autoimmune thrombocytopenic purpura	☐	☐
Pernicious anemia	☐	☐
Myasthenia gravis	☐	☐
Multiple sclerosis	☐	☐
Guillain-Barré syndrome	☐	☐
Stiff-man syndrome	☐	☐
Acute rheumatic fever	☐	☐
Sympathetic ophthalmia	☐	☐
Goodpasture's syndrome	☐	☐
Active Rheumatoid Arthritis	☐	☐

Research Nurse/Investigator Signature:________________________________Date: ____________

References

1. Melero I, Hervas-Stubbs S, Glennie M, Pardoll DM and Chen L.Immunostimulatory monoclonal antibodies for cancer therapy. Nat Rev Cancer 2007; 7: 95-106.

2. Fong L and Small EJ. Anti-cytotoxic T-lymphocyte antigen-4 antibody: the first in an emerging class of immunomodulatory antibodies for cancer treatment. J Clin Oncol 2008; 26: 5275-83.

3. Peggs KS, Quezada SA, Chambers CA, Korman AJ and Allison JP. Blockade of CTLA-4 on both effector and regulatory T cell compartments contributes to the antitumor activity of anti-CTLA-4 antibodies. J Exp Med 2009; 206: 1717-25.

4. Curran MA, Montalvo W, Yagita H and Allison JP. PD-1 and CTLA-4 combination blockade expands infiltrating T cells and reduces regulatory T and myeloid cells within B16 melanoma tumors. Proc Natl Acad Sci U S A 2010; 107: 4275-80.

5. Brahmer JR, Drake CG, Wollner I, Powderly JD, Picus J, Sharfman WH, et al. Phase I study of single-agent anti-programmed death-1 (MDX-1106) in refractory solid tumors: safety, clinical activity, pharmacodynamics, and immunologic correlates. J Clin Oncol 2010; 28: 3167-75.

6. Weber J. Immune checkpoint proteins: a new therapeutic paradigm for cancer--preclinical background: CTLA-4 and PD-1 blockade. Semin Oncol 2010; 37: 430-9.

7. Hodi FS, O'Day SJ, McDermott DF, Weber RW, Sosman JA, Haanen JB, et al. Improved survival with ipilimumab in patients with metastatic melanoma. N Engl J Med 2010; 363: 711-23.

8. Brahmer JR, Tykodi SS, Chow LQ, Hwu WJ, Topalian SL, Hwu P, et al. Safety and activity of anti-PD-L1 antibody in patients with advanced cancer. N Engl J Med 2012; 366: 2455-65.

9. Topalian SL, Hodi FS, Brahmer JR, Gettinger SN, Smith DC, McDermott DF, et al. Safety, activity, and immune correlates of anti-PD-1 antibody in cancer. N Engl J Med 2012; 366: 2443-54.

10. Berger R, Rotem-Yehudar R, Slama G, Landes S, Kneller A, Leiba M, et al. Phase I safety and pharmacokinetic study of CT-011, a humanized antibody interacting with PD-1, in patients with advanced hematologic malignancies. Clin Cancer Res 2008; 14: 3044-51.

11. Sznol M, Hodi FS, Margolin K, McDermott DF, Ernstoff MS, Kirkwood JM, et al. Phase I study of BMS-663513, a fully human anti-CD137 gonist monoclonal antibody, in patients (pts) with advanced cancer (CA). J Clin Oncol 2008; 26: May 20 suppl; abstr 3007.

12. Kjaergaard J, Tanaka J, Kim JA, Rothchild K, Weinberg A and Shu S. Therapeutic efficacy of OX-40 receptor antibody depends on tumor immunogenicity and anatomic site of tumor growth. Cancer Res 2000; 60: 5514-21.

13. Weinberg AD, Rivera MM, Prell R, Morris A, Ramstad T, Vetto JT, et al. Engagement of the OX-40 receptor in vivo enhances antitumor immunity. J Immunol 2000; 164: 2160-9.

14. Gough MJ, Ruby CE, Redmond WL, Dhungel B, Brown A and Weinberg AD. OX40 agonist therapy enhances CD8 infiltration and decreases immune suppression in the tumor. Cancer Res 2008; 68: 5206-15.

15. Piconese S, Valzasina B and Colombo MP. OX40 triggering blocks suppression by regulatory T cells and facilitates tumor rejection. J Exp Med 2008; 205: 825-39.

16. American CAncer Society. Cancer Facts and Figures 2008. Altanta, GA:USA. American Cancer Society; 2008.

17. Chaturvedi AK, Engels EA, Anderson WF, Gillison ML. Incidence trends for human papillomavirus-related and –unrelated oral squamous cell carcinomas in the United States. J Clin Oncol 2008;26:612-619

18. Vermorken et al. (2008). Platinum-based chemotherapy plus cetuximab in head and neck cancer. N Engl J Med. 359:1116-1127.

19. Smith et al. Utilization of free tissue transfer in head and neck surgery. Otolaryngol Head and Neck Surg. 2007;137:182-191.

20. Vergeer et al. Intensity-modulated radiotherapy reduces radiation-induced morbidity and improves health-related quality of life: results of a nonrandomized prospective study using a standardized follow-up program. Int J Radiat Oncol Biol Phys. 2009;74:1-8.

21. D'Souza G, Kreimer AR, Viscidi R, Pawlita M, Fakhry C, Koch WM, Westra WH, Gillison ML. Case-control study of human papillomavirus and oropharyngeal cancer. N Engl J Med. 2007 May 10;356(19):1944-1956.

22. Ang, K. K. et al. Human papillomavirus and survival of patients with oropharyngeal cancer. N. Engl. J. Med. 2010;363, 24–35.

23. O'Rorke et al. Human papillomavirus related head and neck cancer survival: a systemic review and meta-analysis. Oral Oncol. 2012;48:1191-1201.

24. Howlader N, Noone AM, Krapcho M, Neyman N, Aminou R, Altekruse SF, Kosary CL, Ruhl J, Tatalovich Z, Cho H, Mariotto A, Eisner MP, Lewis DR, Chen HS, Feuer EJ, Cronin KA (eds). SEER Cancer Statistics Review, 1975-2009 (Vintage 2009 Populations), National Cancer Institute. Bethesda, MD, http://seer.cancer.gov/csr/1975_2009_pops09/, based on November 2011 SEER data submission, posted to the SEER web site, 2012.

25. Spanos et al. Immune response during therapy with cisplatin or radiation for human papillomavirus-related head and neck cancer. Arch Otolaryngol Head Neck Surg. 2009;135:1137-1146.

26. Ascierto PA, Capone M, Urba WJ, Bifulco CB, et al. The additional facet of immunoscore: immunoprofiling as a possible predictive tool for cancer treatment. J Transl Med. 2013;11:54

27. Pages F, et al. Effector memory t cells, early metastasis, and survival in colorectal cancer. N. Engl. J. Med. 2005

28. Galon J, Pagès F, Marincola FM, Thurin M, Trinchieri G, Fox BA, Gajewski TF, Ascierto PA. The immune score as a new possible approach for the classification of cancer. J Transl Med. 2012 Jan 3; 10():1.

29. Galon J, Costes A, Sanchez-Cabo F, Kirilovsky A, Mlecnik B, Lagorce-Pagès C, Tosolini M, Camus M, Berger A, Wind P, Zinzindohoué F, Bruneval P, Cugnenc PH, Trajanoski Z, Fridman WH, Pagès F. Type, density, and location of immune cells within human colorectal tumors predict clinical outcome. Science. 2006;313:1960–1964.

30. Mlecnik B, Tosolini M, Kirilovsky A, Berger A, Bindea G, Meatchi T, Bruneval P, Trajanoski Z, Fridman WH, Pagès F, Galon J. Histopathologic-based prognostic factors of colorectal cancers are associated with the state of the local immune reaction. J Clin Oncol. 2011;29:610–618.

31. Pagès F, Kirilovsky A, Mlecnik B, Asslaber M, Tosolini M, Bindea G, Lagorce C, Wind P, Marliot F, Bruneval P, Zatloukal K, Trajanoski Z, Berger A, Fridman WH, Galon J. In situ cytotoxic and memory T cells predict outcome in patients with early-stage colorectal cancer. J Clin Oncol. 2009;27:5944–5951.

32. Galon J, Pagès F, Marincola FM, Angell HK, Thurin M, Lugli A, et al. Cancer classification using the Immunoscore: a worldwide task force.J Transl Med. 2012 Oct 3;10:205. doi: 10.1186/1479-5876-10-205.

33. Fox BF, Bell RB. Unpublished data.

34. Wansom D, Light E, Thomas D, et al. Infiltrating lymphocytes and human papillomavirus-16-associated oropharyngeal cancer. Laryngoscope. 2012;122(1):121-127.

35. Turksma AW, Braakhuis BJM, Bloemena E, et al. Immunotherapy for head and neck cancer patients: shifting the balance. Immunotherapy. 2013;5(1):49-61.

36. Albers, A. E., Strauss, L., Liao, T., Hoffmann, T. K. & Kaufmann, A. M. T cell-tumor interaction directs the development of immunotherapies in head and neck cancer. Clinical and Developmental Immunology 2710, 236378.

28. Czystowska, M. et al. The Immune Signature of CD8+CCR7+ T Cells in the Peripheral Circulation Associates with Disease Recurrence in Patients with HNSCC. Clin. Cancer Res. 19, 889–899 (2013).

39. Correale, P. et al. Tumor infiltration by T lymphocytes expressing chemokine receptor 7 (CCR7) is predictive of favorable outcome in patients with advanced colorectal carcinoma. Clin. Cancer Res. 18, 850–857 (2012).

40. Thurlow, J. K. et al. Spectral clustering of microarray data elucidates the roles of microenvironment remodeling and immune responses in survival of head and neck squamous cell carcinoma. Journal of Clinical Oncology 28, 2881–2888 (2010).

41. Distel, L. V. et al. Tumour infiltrating lymphocytes in squamous cell carcinoma of the oro- and hypopharynx: prognostic impact may depend on type of treatment and stage of disease. Oral Oncol. 45, e167–74 (2009).

42. Green, V. L., Michno, A., Stafford, N. D. & Greenman, J. Increased prevalence of tumour infiltrating immune cells in oropharyngeal tumours in comparison to other subsites: relationship to peripheral immunity. Cancer Immunol. Immunother. (2013). doi:10.1007/s00262-013-1395-9

43. Lechner, M. G. et al. Functional characterization of human Cd33+ and Cd11b+ myeloid-derived suppressor cell subsets induced from peripheral blood mononuclear cells co-cultured with a diverse set of human tumor cell lines. J Transl Med 9, 90 (2011).

44. Chikamatsu, K. et al. Immunosuppressive activity of CD14+ HLA-DR- cells in squamous cell carcinoma of the head and neck. Cancer Sci. 103, 976–983 (2012).

45. Vasquez-Dunddel, D. et al. STAT3 regulates arginase-I in myeloid-derived suppressor cells from cancer patients. J. Clin. Invest. (2013). doi:10.1172/JCI60083

46. Albesiano, E. et al. Immunologic consequences of signal transducers and activators of transcription 3 activation in human squamous cell carcinoma. Cancer Res. 70, 6467–6476 (2010).

47. Zitvogel, L., Kepp, O. & Kroemer, G. Immune parameters affecting the efficacy of chemotherapeutic regimens. Nat Rev Clin Oncol 8, 151–160 (2011).

48. Mattarollo, S. R. et al. Pivotal role of innate and adaptive immunity in anthracycline chemotherapy of established tumors. Cancer Res. 71, 4809–4820 (2011).

49. Baum PR, Gayle RB, 3rd, Ramsdell F, Srinivasan S, Sorensen RA, Watson ML, et al. Molecular characterization of murine and human OX40/OX40 ligand systems: identification of a human OX40 ligand as the HTLV-1-regulated protein gp34. Embo J. 1994;13(17):3992-4001.

50. Redmond WL, Weinberg AD. Targeting OX40 and OX40L for the treatment of autoimmunity and cancer. Crit Rev Immunol. 2007;27(5):415-36.

51. Gramaglia I, Weinberg AD, Lemon M, Croft M. Ox-40 ligand: a potent costimulatory molecule for sustaining primary CD4 T cell responses. J Immunol. 1998;161(12):6510-7.

52. Weinberg AD, Wallin JJ, Jones RE, Sullivan TJ, Bourdette DN, Vandenbark AA, et al. Target organ-specific up-regulation of the MRC OX-40 marker and selective production of Th1 lymphokine mRNA by encephalitogenic T helper cells isolated from the spinal cord of rats with experimental autoimmune enciphalomyelitis. Journal of Immunology. 1994;152:4712-21.

53. Weinberg AD, Rivera MM, Prell R, Morris A, Ramstad T, Vetto JT, et al. Engagement of the OX-40 receptor in vivo enhances antitumor immunity. J Immunol. 2000;164(4):2160-9.

54. Buenafe AC, Weinberg AD, Culbertson NE, Vandenbark AA, Offner H. V beta CDR3 motifs associated with BP recognition are enriched in OX-40+ spinal cord T cells of Lewis rats with EAE. J Neurosci Res. 1996;44(6):562-7.

55. Weinberg AD, Lemon M, Jones AJ, Vainiene M, Celnik B, Buenafe AC, et al. OX-40 antibody enhances for autoantigen specific V beta 8.2+ T cells within the spinal cord of Lewis rats with autoimmune encephalomyelitis. J Neurosci Res. 1996;43(1):42-9.

56. Weinberg AD. OX40: targeted immunotherapy - implications for tempering autoimmunity and enhancing vaccines. Trends in Immunology. 2002;23(2):102-9.

57. Gough MJ, Ruby CE, Redmond WL, Dhungel B, Brown A, Weinberg AD. OX40 agonist therapy enhances CD8 infiltration and decreases immune suppression in the tumor. Ca Res 2008;68(13):5206-15.

58. Kjaergaard J, Peng L, Cohen PA, Drazba JA, Weinberg AD, Shu S. Augmentation versus inhibition: effects of conjunctional OX-40 receptor monoclonal antibody and IL-2 treatment on adoptive immunotherapy of advanced tumor. J Immunol. 2001;167(11):6669-77.

59. Kjaergaard J, Tanaka J, Kim JA, Rothchild K, Weinberg A, Shu S. Therapeutic efficacy of OX-40 receptor antibody depends on tumor immunogenicity and anatomic site of tumor growth. Cancer Res. 2000;60(19):5514-21.

60. Lutz E, Wolpoe, M., Ercolini, A., Ladle, B. Weinberg, A., Scheonberger, S., Allison, J, Emens, L., Jaffee, E., Reilly, R.T., editor. Effects of CTLA-4, CD40, and OX-40 targeted immune modulation on antitumor immunity in HER-2/neu mouse model of breast cancer. AACR; 2002; San Francisco.

61. Kaleeba JA, Offner H, Vandenbark AA, Lublinski A, Weinberg AD. The OX-40 receptor provides a potent co-stimulatory signal capable of inducing encephalitogenicity in myelin-specific CD4+ T cells. Int Immunol. 1998;10(4):453-61.

62. Chambers CA, Kuhns MS, Egen JG, Allison JP. CTLA-4-mediated inhibition in regulation of T cell responses: mechanisms and manipulation in tumor immunotherapy. Annu Rev Immunol. 2001;19:565-94.

63. Weinberg AD, Vella AT, Croft M. OX-40: life beyond the effector T cell stage. Semin Immunol. 1998;10(6):471-80.

64. Redmond WL, Gough MJ, Charbonneau B, Ratliff TL, Weinberg AD. Defects in the acquisition of CD8 T cell effector function after priming with tumor or soluble antigen can be overcome by the addition of an OX40 agonist. J Immunol. 2007;179(11):7244-53.

65. Prell RA, Evans DE, Thalhofer C, Shi T, Funatake C, Weinberg AD. OX40-mediated memory T cell generation is TNF receptor-associated factor 2 dependent. J Immunol. 2003;171(11):5997-6005.

66. Lee SW, Park Y, Song A, Cheroutre H, Kwon BS, Croft M. Functional dichotomy between OX40 and 4-1BB in modulating effector CD8 T cell responses. J Immunol. 2006;177(7):4464-72.

67. Huddleston CA, Weinberg AD, Parker DC. OX40 (CD134) engagement drives differentiationof CD4(+) T cells to effector cells. Eur J Immunol. 2006;36(5):1093-103.

68. Lathrop SK, Huddleston CA, Dullforce PA, Montfort MJ, Weinberg AD, Parker DC. A signal through OX40 (CD134) allows anergic, autoreactive T cells to acquire effector cell functions. J Immunol. 2004;172:6735-43.

69. Valzasina B, Guiducci C, Dislich H, Killeen N, Weinberg AD, Colombo MP. Triggering of OX40 (CD134) on CD4(+)CD25+ T cells blocks their inhibitory activity: a novel regulatory role for OX40 and its comparison with GITR. Blood. 2005;105(7):2845-51.

70. Cepowicz D, Zareba K, Gryko M, Stasiak-Bermuta A, Kedra B. Determination of the activity of CD134 (OX-40) and CD137 (4-1BB) adhesive molecules by means of flow cytometry in patients with colorectal cancer metastases to the liver. Pol Przegl Chir. 2011;83(8):424-9.

71. Curti BD, Kovacsovics-Bankowski M, Morris N, Walker E, Chisholm L, et al. OX40 is a potent immune-stimulating target in late stage cancer patients. Cancer Res. 2013 Nov 26 (Epub ahead of print)

72. Gough MJ, Crittenden MR, Sarff M, Pang P, Seung SK, Vetto JT, et al. Adjuvant therapy with agonistic antibodies to CD134 (OX40) increases local control after surgical or radiation therapy of cancer in mice. J Immunother. 2010;33(8):798-809.

73. Carthon BC, Wolchok JD, Yuan J, Kamat A, Ng Tang DS, Sun J, et al. Preoperative CTLA-4 blockade: tolerability and immune monitoring in the setting of a presurgical clinical trial. Clin Cancer Res. 2010;16(10):2861-71.

74. Hirsch DL, Bell RB, Dierks EJ, Potter JK, Potter BE. Analysis of microvascular free flaps for reconstruction of advanced mandibular osteoradionecrosis: a retrospective cohort study. J Oral Maxillofac Sur. 2008 Dec;66(12):2545-56

75. Arce K, Bell RB, Potter JK, Buehler MJ, Potter BE, Dierks EJ. Vascularized free tissue transfer for reconstruction of ablative defects in oral and oropharyngeal cancer patients undergoing salvage surgery following concomitant chemoradiation. Int J Oral Maxillofac Surg. 2012 Jun;41(6):733-8

76. Schreiber, R. D., Old, L. J. & Smyth, M. J. Cancer Immunoediting: Integrating Immunity's Roles in Cancer Suppression and Promotion. *Science* 331, 1565–1570 (2011).

77. Clavien PA, Barkun J, de Oliverira ML, Vauthey JN, Dindo D, Schulick RD, de Santibanes E, Pekolj, Slankamenac K, Bassi C, Graf R, Vonlanthen R, Padbury R, Cameron JL, Makuuchi M. Ann Surg. 2009 Aug;250(2):187-96
