# Supplementary material for: Neoadjuvant anti-OX40 (MEDI6469) therapy in patients with head and neck squamous cell carcinoma activates and expands antigen-specific tumor-infiltrating T cells
Source: Nat Commun. 2021 Feb 16;12:1047. doi: 10.1038/s41467-021-21383-1 (PMC7886909; doi:10.1038/s41467-021-21383-1)
Supplement: Supplementary file 7 — Reporting Summary [file 41467_2021_21383_MOESM7_ESM.pdf]

nature research

Corresponding author(s): Rebekka Duhen & Andrew D. Weinberg

Last updated by author(s): Oct 29, 2020

Reporting Summary

Nature Research wishes to improve the reproducibility of the work that we publish. This form provides structure for consistency and transparency in reporting. For further information on Nature Research policies, see Authors & Referees and the Editorial Policy Checklist .

Statistics

For all statistical analyses, confirm that the following items are present in the figure legend, table legend, main text, or Methods section.

n/a	Confirmed	
☐	☒	The exact sample size (*n*) for each experimental group/condition, given as a discrete number and unit of measurement
☐	☒	A statement on whether measurements were taken from distinct samples or whether the same sample was measured repeatedly
☐	☒	The statistical test(s) used AND whether they are one- or two-sided<br>*Only common tests should be described solely by name; describe more complex techniques in the Methods section.*
☒	☐	A description of all covariates tested
☐	☒	A description of any assumptions or corrections, such as tests of normality and adjustment for multiple comparisons
☐	☒	A full description of the statistical parameters including central tendency (e.g. means) or other basic estimates (e.g. regression coefficient) AND variation (e.g. standard deviation) or associated estimates of uncertainty (e.g. confidence intervals)
☐	☒	For null hypothesis testing, the test statistic (e.g. *F*, *t*, *r*) with confidence intervals, effect sizes, degrees of freedom and *P* value noted<br>*Give P values as exact values whenever suitable.*
☒	☐	For Bayesian analysis, information on the choice of priors and Markov chain Monte Carlo settings
☒	☐	For hierarchical and complex designs, identification of the appropriate level for tests and full reporting of outcomes
☒	☐	Estimates of effect sizes (e.g. Cohen's *d*, Pearson's *r*), indicating how they were calculated

Our web collection on statistics for biologists contains articles on many of the points above.

Software and code

Policy information about availability of computer code

Data collection	For cell acquisition and cell sorting, data were collected at the Flow Cytometry Core at the EACRI using a FACSAriaII SORP, LSRII and LSR Fortessa with FACSDiva software version 8.0.2. For use with the Attune Flow Cytometer, Attune Nxt Flow Cytometer Software version 3.1.1243.0 was used. High throughput sequencing was performed using the Illumina Miseq (for TCRsequencing) and the Illumina HiSeq (for Whole Exome sequencing) at the Molecular Genomics Laboratory.<br>Digital images were captured with the PerkinElmer Vectra-Polaris platform.
Data analysis	Software and code used in this study are all described in methods. Included are the somatic single nucleotide variant calling algorithms MuTect (version 1.1.7) , Somaticsniper (version 1.0.5.0) , Strelka (version 1.0.15) and Varscan (version 2.3.6) and the open source server netMHCpan 4.0 for HLA classI peptide binding predictions. RNA alignment was performed using STAR version 2.5.3a, duplicate reads were marked using Mark Duplicate function in Picard tools version 2.6.0, and fragments per kb per million mapped reads (FPKM) values were calculated using cufflinks 2.2.1.<br><br>FlowJo version 10.6.0 and version 9.9.6 were used for analysis of flow cytometric data, GraphPad Prism version 8 was used for statistical analysis and the ImmunoSEQ Analyzer Platform (version 3.0) from Adaptive Biotechnologies for the analysis of the TCRsequencing data. Multiplexed images were analyzed with InForm Software (version 2.4.2).

For manuscripts utilizing custom algorithms or software that are central to the research but not yet described in published literature, software must be made available to editors/reviewers. We strongly encourage code deposition in a community repository (e.g. GitHub). See the Nature Research guidelines for submitting code & software for further information.

Data

Policy information about availability of data

All manuscripts must include a data availability statement. This statement should provide the following information, where applicable:

- Accession codes, unique identifiers, or web links for publicly available datasets
- A list of figures that have associated raw data
- A description of any restrictions on data availability

TCR sequencing data has been deposited in the ImmuneACCESS database with the following accession code (DOI 10.21417/RD2020NC and URL clients.adaptivebiotech.com/pub/duhen-2020-nc). Processed sequencing data and predicted neoantigens are available in the supplementary data 2, supporting statistical documentation is summarized in supplementary data 3.

Field-specific reporting

Please select the one below that is the best fit for your research. If you are not sure, read the appropriate sections before making your selection.

☒ Life sciences ☐ Behavioural & social sciences ☐ Ecological, evolutionary & environmental sciences

For a reference copy of the document with all sections, see nature.com/documents/nr-reporting-summary-flat.pdf

Life sciences study design

All studies must disclose on these points even when the disclosure is negative.

Sample size	As this study was a Phase I safety study, a sample size calculation based on the formal statistical testing was not performed.<br>19 patients signed consent, 2 of which screen-failed, thus 17 patients were treated. The primary objective of this trial was to examine the safety and feasibility of definitive surgical resection following MEDI6469 administration, which was determined with a 6 patient safety lead-in with early stopping rules based upon unacceptable toxicity defined as grade 4 or 5 toxicity. Based upon our initial phase I trial in the metastatic setting in which grade 4-5 toxicity occurred in 1/28 patients, we assumed that any delay in surgery related to OX40 administration in the first 6 patients would be significant and terminate the trial. The overall sample size was based upon immunological parameters. The number of patients enrolled in the time-course portion of the trial (n=9) was based upon the observation that any significant change in effector or memory immune cell populations would be considered adequate to determine potential immune effect and inform the relative timing of peak immune response to anti-OX40. For 2-sided t-tests of tumor tissue, tissue at tumor margins, blood samples, and lymph node samples in the treatment samples and historical controls, a sample size of 32 provides greater than 80% power to detect a 1.5 fold difference in group means of cell counts, such as Tregs and effector cells and others described in Section 9.1 of the protocol, assuming a coefficient of variation of .55 as observed in the Tregs of the phase 1 trial (Curti et al, Cancer Res, 2013). The second stage was designed to include an expansion cohort of up to 21 additional patients in the cohort selected, based on the most promising immune response in peripheral blood and tumors of patients enrolled in stage I.
Data exclusions	HNOX02 and HNOX12 were excluded from the study (prior to treatment):<br>As detailed in Section 4.2 of the study protocol, exclusion criteria included active infection; active autoimmune disease including patients with inflammatory bowel disease as determined by an autoimmune questionnaire; Previous treatment with mouse monoclonal antibodies; Need for chronic maintenance oral steroids > 5mg prednisone daily equivalent; active infection; or any medical or psychiatric condition that in the opinion of the PI would preclude compliance with study procedures.<br><br>HNOX02 had pre-existing superficial venous thrombosis of RLE indicative of mechanical clotting risk, as well as the risk of nephrotoxicity in the setting of DM, stage III CKD (eGFR = 58) and HTN. These risks outweighed theoretical benefit of study, and following DPARQ conference, he withdrew from the trial.<br><br>HNOX012 = was found to have + Hep C antibody on screening labs<br><br>Multiplex IHC analysis was performed on 15 out of the 17 treated patients. Patient 01 and 08 were excluded due to insufficient / low quality biopsy material (FFPE tissue).
Replication	All experiments have been performed with appropriate replicates as described in the Figures and Methods sections.
Randomization	The first participant was enrolled in a D-26 cohort, after which the time-course portion of the protocol was modified. The next 9 patients were enrolled in D8, D12 and D19 cohorts based on practical design. The next 7 patients were enrolled in the D12 cohort, which was decided based upon immunologic response in the first 9 patients; except for one patient, which was enrolled in D19 due to a delay in surgery.<br>Thus, randomization was not appropriate in this phase I study.
Blinding	For IHC analysis and spot selection, authors involved in data analysis were blinded to group allocation, all clinical (e.g recurrence) and epidemiological data.<br>For phenotyping of PBMC from subjects, investigators were not blinded to group allocation, as surgical samples were collected at different timepoints matching the cohort.

Reporting for specific materials, systems and methods

We require information from authors about some types of materials, experimental systems and methods used in many studies. Here, indicate whether each material, system or method listed is relevant to your study. If you are not sure if a list item applies to your research, read the appropriate section before selecting a response.

Materials & experimental systems

n/a	Involved in the study
☐	☒ Antibodies
☒	☐ Eukaryotic cell lines
☒	☐ Palaeontology
☒	☐ Animals and other organisms
☐	☒ Human research participants
☐	☒ Clinical data

Methods

n/a	Involved in the study
☒	☐ ChIP-seq
☐	☒ Flow cytometry
☒	☐ MRI-based neuroimaging

Antibodies

Antibodies used

Flow Cytometry:
Allophycocyanin (APC)-Cy7 and brilliant violet (BV) 605 anti-CD3 (UCHT1; 1:100—#300426 and #300406, respectively),
BV785 anti-CD4 (OKT-4; 1:200—#317442),
BV510 anti-CD8 (RPA-T8; 1:100—#301048),
PerCP/Cy5.5 anti-CD19 (HIB19; 1:50—#302230),
BV650 anti-CD25 (BC96; 1:100—#302634),
APC and Alexa Fluor (AF) 488 anti-CD38 (HIT2; 1:50—#303510 and #303512, respectively),
AF700 and BV711 anti-CD45RA (HI100; 1:50—#304120 and 304137, respectively),
BV605 anti-CD69 (FN50; 1:50—#310938),
BV421 anti-CD127 (A019D5; 1:50—#351310),
PE-Cy7 anti-PD-1 (EH12.2H7; 1:50—#329918),
PE-Cy7 anti-4-1BB (4B4-1; 1:40—#309818),
PE/Dazzle 594 anti-CCR7 (G043H7; 1:50—#353236),
BV711 anti-HLA-DR (L243; 1:100—#307644),
BV510 anti-IgD (IA6-2; 1:50—#348220) (all from Biolegend);

PE-CF594 anti-CD24 (ML5; 1:50—#562405),
BV421 anti-CD27 (M-T271; 1:50—#562513),
FITC anti-CD127 (HIL-7R-M21; 1:10—#560549),
PE anti-OX40 (ACT35; 1:40—#555838),
PE-Cy7 anti-PD-1 (EH12.1; 1:50—#561272),
PE anti-granzyme B (GB11; 1:200—#561142),
AF 488 and PE anti-Ki-67 (B56; 1:100—#561165 and 1:50 #556027) (all from BD Biosciences);

APC-efluor780 anti-CD8 (RPA-T8; 1:100—#47-0088-42),
APC and PE-Cy7 anti-CD39 (eBioA1; 1:100—#17-0399-42 and #25-0399-42, respectively),
PE and PerCP-efluor710 anti-CD103 (B-Ly7 and Ber-ACT8; 1:100—#12-1038-42 and 1:50—#46-1037-42, respectively),
efluor450 and AF700 anti-Foxp3 (PCH101; 1:40—#48-4776-41 and 1:25—#56-4776-41, respectively),
biotinylated anti-ICOS (ISA-3; 1:100—#13-9948-82),
Streptavidin APC-efluor780 (1:100—#47-4317-82) (all from eBioscience).

murine IgG1 anti-OX40 antibody (clone 9B12) was produced, purified and provided by AstraZeneca (MedImmune) and administered at 0.4mg/kg at days 1, 3 and 5 prior to surgery.

IHC:
FOXP3 (236A/E7; 1:100), Abcam #ab20034
PD-L1 (E1L3N; 1:250), Cell Signaling # 13684S
CD8 (SP16; 1:50), Abcam #ab101500
CD3 (SP7; 1:50), Abcam #ab16669
CD163 (MRQ-26; Pre-diluted), Roche Tissue Diagnositcs #760-4437
CK (AE1/AE3; 1:100), Agilent DAKO #GA05361-2
CD103 (EPR4166(2); 1:2000), Abcam #ab129202
Ki-67 (D2H10; 1:50), Cell Signaling #9027

Validation

The activity and specificity of the 9B12 antibody was validated by AstraZeneca (MedImmune) for therapeutic treatment of cancer

patients.
For Flow cytometry antibodies, each antibody lot received from the manufacturer was validated in-house on healthy donor PBMC samples or stimulated PBMC samples to validate the specificity and dilution of respective antibodies used in this study.

For IHC antibodies, listed below are the references for each antibody and validated applications:
Foxp3 clone 236A/E7: https://www.abcam.com/foxp3-antibody-236ae7-ab20034.html
suitable for IHC-P (10 ug/ml), WB (4-5 ug/ml) and ICC/IF (assay dependent)
PD-L1 clone E1L3N: https://www.cellsignal.com/products/primary-antibodies/pd-l1-e1l3n-xp-rabbit-mab/13684?Ntk=Products&Ntt=13684
suitable for WB (1:1000), IP (1:50), IHC-BOND (1:400), IHC-P (1:200) and Flow Cyt (1:400)
CD8 clone SP16: https://www.abcam.com/cd8-alpha-antibody-sp16-ab101500.html
suitable for IHC-P (1:100), Flow-Cyt (1:1000)
CD3 clone SP7: https://www.abcam.com/cd3-antibody-sp7-ab16669.html
suitable for Flow Cyt (1:1000), IHC-P (1:150), WB (1:25), mIHC (assay dependent)
CD163 clone MRQ-26: https://www.cellmarque.com/antibodies/CM/2010/CD163_MRQ-26
suitable for IHC-P in-vitro diagnostic (pre-diluted)
CK clone AE1/AE3: https://www.agilent.com/store/en_US/Prod-GA05361-2/GA05361-2
suitable for IHC-P, in-vitro diagnostic (ready-to-use)
CD103 clone EPR4166(2): https://www.abcam.com/cd103-antibody-epr41662-ab129202.html
suitable for IHC-P (1:500 - 1:1000)
Ki-67 clone D2H10: https://www.cellsignal.com/products/primary-antibodies/ki-67-d2h10-rabbit-mab-ihc-specific/9027?Ntk=Products&Ntt=9027
suitable for IHC-P (1:400)

Human research participants

Policy information about studies involving human research participants

Population characteristics

Characteristics of all participants is summarized in supplementary table1 in the manuscript and below:
Age (years), Mean ± SD 60 ± 9

Sex, n (%)
Men 13 (76)
Women 4 (24)

HPV status, n
positive 6
negative 11

AJCC (7th Edition) clinical stage, n (%)
II 2 (12)
III 5 (29)
IV/IV-A 10 (59)

AJCC (8th Edition) clinical stage, n (%)
I 3 (18)
II 4 (23.5)
III 6 (35)
IV/IV-A 4 (23.5)

Site, n (%)
Oral 7 (41)
Oropharynx 7 (41)
Hypopharynx 1 (6)
Larynx 2 (12)

Prior Therapy, n (%)
Treatment naïve 14 (82)
Radiation Therapy 3 (18)

Recruitment

The participants were recruited with no self-selection bias and inclusion criteria are detailed in Section 4.1. of the study protocol. Importantly, any previously untreated patient with HNSCC was a candidate for inclusion. Therefore, there was potential for a selection bias to patients that were medically fit or who had less aggressive disease biology. While all patients had locally advanced disease based upon the presence or absence of lymph node metastasis or bone invasion, it is possible that immune responses could vary depending upon tumor heterogeneity and disease burden.

Here are the guidelines that were followed for Patient Recruitment at the EACRI, Portland, OR:

	A. Provider Referral- Physicians, Nurse Practitioners, Nurse Navigators, etc. may contact the clinical research office in search of trial availability for their patients. They provide a brief description of the disease and treatment history and research staff will then inform them of any potential trials for which the patient may qualify.<br>B. Patient Self-Referral- Patients may contact the clinical research office in search of trial availability. Patient will collect their own treatment details and provide to research staff. Research staff will then inform them of any potential trials for which they may qualify and coordinate a physician visit.<br>C. New Patient Review- Research staff may briefly review patient charts at Providence-affiliated clinics to determine if any trials are available for the patient and will then inform the Treating Physician as applicable.<br>D. Tumor Boards- These conferences occur allowing Physicians of different specialties to conduct complete patient reviews and collaboratively discuss best available treatment options. Research staff may attend such conferences and voice trial options when they are available.<br>E. Cancer Institute Website- A list of open clinical trials is posted on the Cancer Institute website and provides general study information for patients and providers.<br>F. Recruitment Material- pamphlets, posters, videos, etc. may be utilized in clinics or sent to providers. These materials may be provided by the study sponsor or created by our research team. All patient-facing items will be IRB approved.
Ethics oversight	Protocol 14-042 was approved by the Institutional Review Board at Providence Health and Services - Oregon. The study was designed and the manuscript was written by the authors, who are responsible for the accuracy of its content. The trial was conducted in accordance with the ethical principles of the Declaration of Helsinki and with adherence to the Good Clinical Practice guidelines, as defined by the International Conference on Harmonization. All patients provided written informed consent.

Note that full information on the approval of the study protocol must also be provided in the manuscript.

Clinical data

Policy information about clinical studies

All manuscripts should comply with the ICMJEguidelines for publication of clinical research and a completedCONSORT checklist must be included with all submissions.

Clinical trial registration	NCT02274155
Study protocol	The study protocol is included in the manuscrip at supplementary data.
Data collection	Patients were enrolled following IRB approval, between December 2014 until April 2017. A prebiospsy specimen and a day of surgery specimen was collected for each patient, together with peripheral blood. Peripheral blood mononuclear cells were also collected at D34 and D55 after surgery as follow-up. Blood draws and surgery took place at Providence Portland Medical Center, Portland Oregon and data were collected by the EACRI clinical trials office.
Outcomes	The primary endpoint in this phase I safety study was delay in time to surgery; secondary endpoints included exploratory assessments to determine the timing of peak immunologic effect and to compare the immunologic phenotypes of TIL subsets before and after anti-OX40 administration.

Flow Cytometry

Plots

Confirm that:

- [x] The axis labels state the marker and fluorochrome used (e.g. CD4-FITC).
- [x] The axis scales are clearly visible. Include numbers along axes only for bottom left plot of group (a 'group' is an analysis of identical markers).
- [x] All plots are contour plots with outliers or pseudocolor plots.
- [x] A numerical value for number of cells or percentage (with statistics) is provided.

Methodology

Sample preparation	PBMC and tumor tissue were received prior to and after anti-OX40 treatment. Tumor tissue was enzymatically digested and lymphoctyes were enriched by FICOLL separation. Samples were cryopreserved and thawed prior to flow cytometric analysis or cell sorting. For TCRb sequencing, DNA was prepared from sorted cell populations.
Instrument	FACS AriaII (BD Biosciences) was used for single cell sorting; BD LSRFortessa, LSRII (BD Biosciences) and the Attune flow cytometer (Thermo Fisher Scientific) were used for flow cytometric analysis of the samples.
Software	FACSDiva software version 8.0.2 was used to acquire flow cytometric data and both FlowJo version 10.6.0 and version 9.9.6 were used for analysis.
Cell population abundance	The purity of the sorted cell populations was above 99%, when analyzing post-sort populations.The frequency of CD4 and CD8, as well as the respective subpopulations (DN, SP and DP cells) varied between samples. For the samples that were analyzed by TCRb sequencing, between 10000-500000 cells were sorted per subset.

Gating strategy

FSC/SSC gates were used to isolate viable, single cells. Live/dead markers were used to exclude dead cells. CD3 was used to enrich for T cells. Gating for CD4 T cells included CD45RA and CCR7 to enrich for memory populations, while excluding CD127-CD25+ T regulatory cells, or alternatively, Foxp+ CD25 cells.

CD8 T cell memory cell subsets were defined by CD45RA and CCR7 and further distinguished in to DN (CD39-/CD103-), SP (CD39-CD103+) and DP (CD39+/CD103+) cells.

Panel 1 and 2 of the immune phenotyping included activation markers such as Ki67, ICOS, PD-1, CD38, which were assessed on gated memory CD4 or CD8 populations.

☒ Tick this box to confirm that a figure exemplifying the gating strategy is provided in the Supplementary Information.
